# deltaRpkm: an R package for a rapid detection of differential gene presence between related bacterial genomes

**DOI:** 10.1186/s12859-019-3234-2

**Published:** 2019-12-02

**Authors:** Hatice Akarsu, Lisandra Aguilar-Bultet, Laurent Falquet

**Affiliations:** 1 Department of Biology, University of Fribourg, Fribourg, Switzerland; 2 Swiss Institute of Bioinformatics, BUGFri group, Fribourg, Switzerland; 3 Institute of Veterinary Bacteriology, Vetsuisse Faculty, University of Bern, Bern, Switzerland; 4 Graduate School for Cellular and Biomedical Sciences, University of Bern, Bern, Switzerland; 5 Currently at the Department of Infectious Diseases and Hospital Epidemiology, University Hospital Basel, Basel, Switzerland

**Keywords:** Comparative genomics, RPKM, Differential gene presence/absence

## Abstract

**Background:**

Comparative genomics has seen the development of many software performing the clustering, polymorphism and gene content analysis of genomes at different phylogenetic levels (isolates, species). These tools rely on de novo assembly and/or multiple alignments that can be computationally intensive for large datasets. With a large number of similar genomes in particular, e.g.*,* in surveillance and outbreak detection, assembling each genome can become a redundant and expensive step in the identification of genes potentially involved in a given clinical feature.

**Results:**

We have developed deltaRpkm, an R package that performs a rapid differential gene presence evaluation between two large groups of closely related genomes. Starting from a standard gene count table, deltaRpkm computes the RPKM per gene per sample, then the inter-group *δRPKM* values, the corresponding median *δRPKM* (*m*) for each gene and the global standard deviation value of *m* (*s*_*m*_). Genes with *m* >  = 2 ∗ *s*_*m*_ (standard deviation *s* of all the *m* values) are considered as “differentially present” in the reference genome group. Our simple yet effective method of differential RPKM has been successfully applied in a recent study published by our group (*N* = 225 genomes of *Listeria monocytogenes*) (Aguilar-Bultet et al. Front Cell Infect Microbiol 8:20, 2018).

**Conclusions:**

To our knowledge, deltaRpkm is the first tool to propose a straightforward inter-group differential gene presence analysis with large datasets of related genomes, including non-coding genes, and to output directly a list of genes potentially involved in a phenotype.

## Background

In comparative genomics the gene presence/absence analysis is commonly performed by multiple alignment calculations on whole genomes or on their subsets as pan-core-genome analysis. Multiple alignment approaches like Mauve [2] and Mugsy [3] become quickly very computationally intensive and unsuitable when dealing with increasing number of genomes. For instance, in the case of *N* = 57 *E.coli* genomes, Mauve run is not finished after 2 days, while Mugsy needs about 20 h (see [3]). Pan-core-genome tools like Microscope [4], Large-Scale Blast Score Ratio (LS-BSR) [5] require genome assembly and gene prediction steps before performing all-against-all Blast calculations. Roary [6] performs a clustering of highly similar sequences before executing all-against-all Blast searches only on these subsets of pre-clustered genes, still requiring the assembly and annotation of all genomes [6]. Bacterial Pan-Genome Analysis tool (BPGA) [7] is fast by clustering the gene sequences like Roary and then aligning them with MUSCLE instead of applying an all-against-all Blast method. Overall, these pan-genome methods run fast on a small scale, e.g.*,* ~ 3 min for BPGA with *N* = 28 *Streptococcus pyogenes* samples (genome size ~ 1.8 Mb) [7] and ~ 6 min for Roary for *N* = 24 *Salmonella enterica, serovar Typhi* samples (genome size ~ 4.8 Mb) [6]. However, none of them is practical for larger datasets, e.g.*,* BPGA takes 7 h for 1000 genomes for 4GB of RAM [7] and Roary produces a pan-genome from 1000 isolates in about 4.5 h, using 13GB of RAM [6]. The above methods are focusing on the protein coding genes, neglecting the non-coding features e.g.*,* small RNA [8]. Other methods like core genome MultiLocus Sequence Typing (cgMLST) are not appropriate for gene presence/absence since the analysis is based on the core-genome, potentially present in all genomes of certain species [9, 10].

Increasing number of studies in human or veterinary clinical genomics, especially those focusing on outbreak detection and tracking, involve a large number of similar genomes to be compared. For such particular cases, we propose a simple yet effective approach using a canonical gene read count table, short-cutting the intensive genome assembly and annotation tasks. Our user-friendly and open-source R package, deltaRpkm, identifies putative genes involved in a given phenotype by inferring their presence/absence from their differential coverage between a reference genome group and a comparison group.

## Implementation

### Input files

**Fig. 1:**
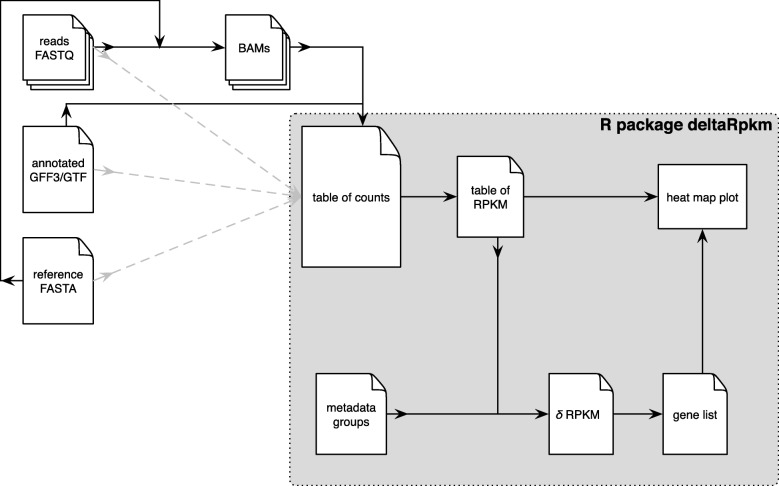
Overview of a deltaRpkm workflow. Black arrows indicate the main pipeline; dotted arrows show an alternative route with STAR. The package is written in R and takes as input a canonical coverage table, plus the design information given by the user as a metadata table. The strength of deltaRpkm relies on bypassing the tedious assembly and annotation steps typical of comparative genomics. Instead, deltaRpkm uses a basic gene read counts table (based on the mapping against a reference genome) to compute inter-group differential RPKM values per gene and outputs a list of candidate genes as present in the samples of the reference genome group (and absent from the comparison group)

The deltaRpkm pipeline requires as input data metadata and gene read count tables. The read count table can be derived from standard methods like bedtools multicov [11] based on a reference genome annotation file and the bam files produced by bwa mem [12]. Alternatively, the rapid RNA-seq aligner STAR can be used to obtain the coverage table [13] (Fig. 1).

### Definition of the phenotypic groups

The analysis is centered around a pairwise comparison of gene differential presence between genomes categorized into two different groups according to a selected phenotype: i) a group 1 that shares the phenotype A of the reference genome and ii) a group 2 that does not have the reference phenotype A. This phenotype information per group is provided in the metadata table. The design of the analysis is given in the deltaRpkm::loadMetadata function that loads the grouping criteria of the dataset based on the metadata information.

### Conversion of gene read counts to RPKM

The pipeline runs the deltaRpkm::rpkm function to normalize raw read counts with the validated RPKM method (Reads Per Kilobase per Million mapped reads), that takes into account sequencing depth and gene length [14]. For a given sample *s* of total read counts *Ns*, the library size correction of read counts (*RPMj*) corresponds to a scaling factor (*scalingFactor*) applied to the reads counts per gene (*readCountsPerGene*), as:

$$ scalingFactor=\frac{N_s}{10^6} $$

$$ {RPM}_j=\frac{readsCountsPerGene}{scalingFactor} $$

Then, for a given gene *j* the *RPKMj* value is computed by weighing in the gene length (*geneLength*):

$$ RPK{M}_j=\frac{RP{M}_j}{geneLength\cdot {10}^{-3}} $$

### Inter-group RPKM values (*δRPKM*)

For each pairwise comparison of the RPKM values of a gene *j* between a genome *x* from group 1 (reference genome) and a genome *y* from group 2, deltaRpkm::deltarpkm function computes the difference of their RPKM values at gene *j (δRPKMj*) as:

$$ \delta RPK{M}_j= RPK{M_j}_x- RPK{M_j}_y $$

### Selection of genes differentially present in the reference group

**Fig. 2:**
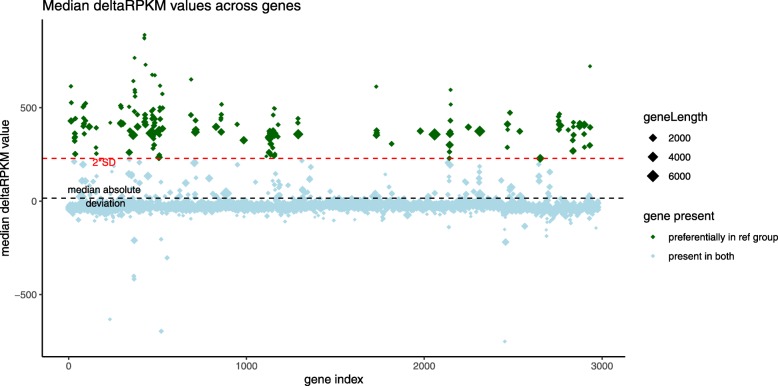
Distribution of the median δRPKM values across all genes. For a given dataset analysis and for a given gene, the median value *m* of all its *δRPKM* is plotted (diamonds). A standard deviation of all the gene median values (*s*_*m*_) is then used to threshold (set as 2 ∗ *s*_*m*_ by default) the significance of differential presence between the two groups of samples. Genes with a median *δRPKM* value *m* >  = 2 ∗ *s*_*m*_ are considered as differentially present in the reference group. The red dotted line corresponds to 2 ∗ *s*_*m*_. The grey dotted line corresponds to the Median Absolute Deviation (MAD). This summary plot can be produced when running the method deltaRpkm::median_plot. A dataset of size *N* = 51 from *Listeria monocytogenes* (genome size ~ 3 Mb for ~ 3 K genes) was used for the analysis represented in the figure, see [1].

The set of genes potentially involved in the selected phenotype correspond to genes that are considered differentially present in the reference genome group, but absent from the comparison group. The deltaRpkm functions to infer those genes are grouped into a main method called deltarpkm::deltaRPKMStats. For each gene *j*, the median value *m*_*j*_ of all its pairwise *δRPKM* values is calculated, followed by the standard deviation *s*_*m*_ of all genes *m* values. Genes with *m* >  = 2 ∗ *s*_*m*_ are considered as present in group 1 of the reference genome and absent from group 2 (Fig. 2). This threshold is relatively stringent and arbitrary, but safer to avoid false positives. Users of deltaRpkm could potentially use the robust Median Absolute Deviation (MAD) as the lower limit to accept a gene differentially present in the reference group. However, this increases the risk of revealing false positives.

### Visualisation of the filtered genes

**Fig. 3:**
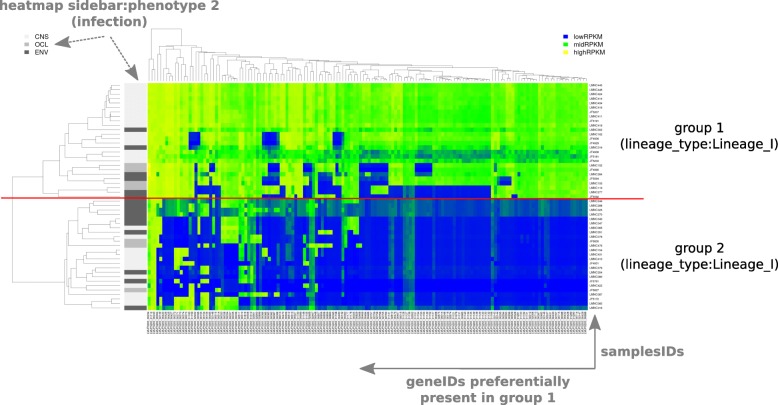
Heatmap of the RPKM distribution of the selected genes. These genes are considered as differentially present between group 1 (samples that have the same phenotype as the reference genome) and group 2 of samples. A dataset of N = 51 of *Listeria monocytogenes* genomes is represented in this figure

For a more visual evaluation of the selected genes potentially involved in the studied phenotype, deltaRpkm provides a plot function called deltarpkm::rpkmHeatmap which is based on gplots::heatmap.2 method (https://CRAN.R-project.org/package=gplots). This deltaRpkm function plots the RPKM values of the selected genes as a heatmap (Fig. 3). The heatmap color scale is based on the boundaries of the RPKM bimodal distribution (Additional file 1: Figure S1).

The different steps and main functions for a quick start with deltaRpkm are summarized in the Table 1.

**Table 1:** Main functions for a differential gene presence/absence analysis with deltaRpkm. Functions are listed in the chronological order of usage

Function name	Description	Output(s)
loadMetadata()	format the user metadata table	data frame of the design table
rpkm()	convert read counts to RPKM	data frame of RPKM values
deltarpkm()	compute pairwise *δRPKM* values (samples from group 1 ~ samples from group 2)	data frame of samples inter-group *δRPKM* values, per gene
deltaRPKMStats()	compute 1) median *δRPKM* values of each gene, 2) global standard deviation of all medians and 3) selection of genes passing a given threshold	data frame with genes annotated as differentially present in reference group 1 versus comparison group 2
median_plot()	diagnostics plot to visualize the median *δRPKM* values of each gene and highlight the selected genes dsitribution	deltaRpkm_medians_plot.pdf file in the working directory
rpkmHeatmap()	heatmap of the RPKM values of the selected set of genes	deltaRpkm_heatmap.tiff file in the working directory

### Tutorial

**Fig. 4:**
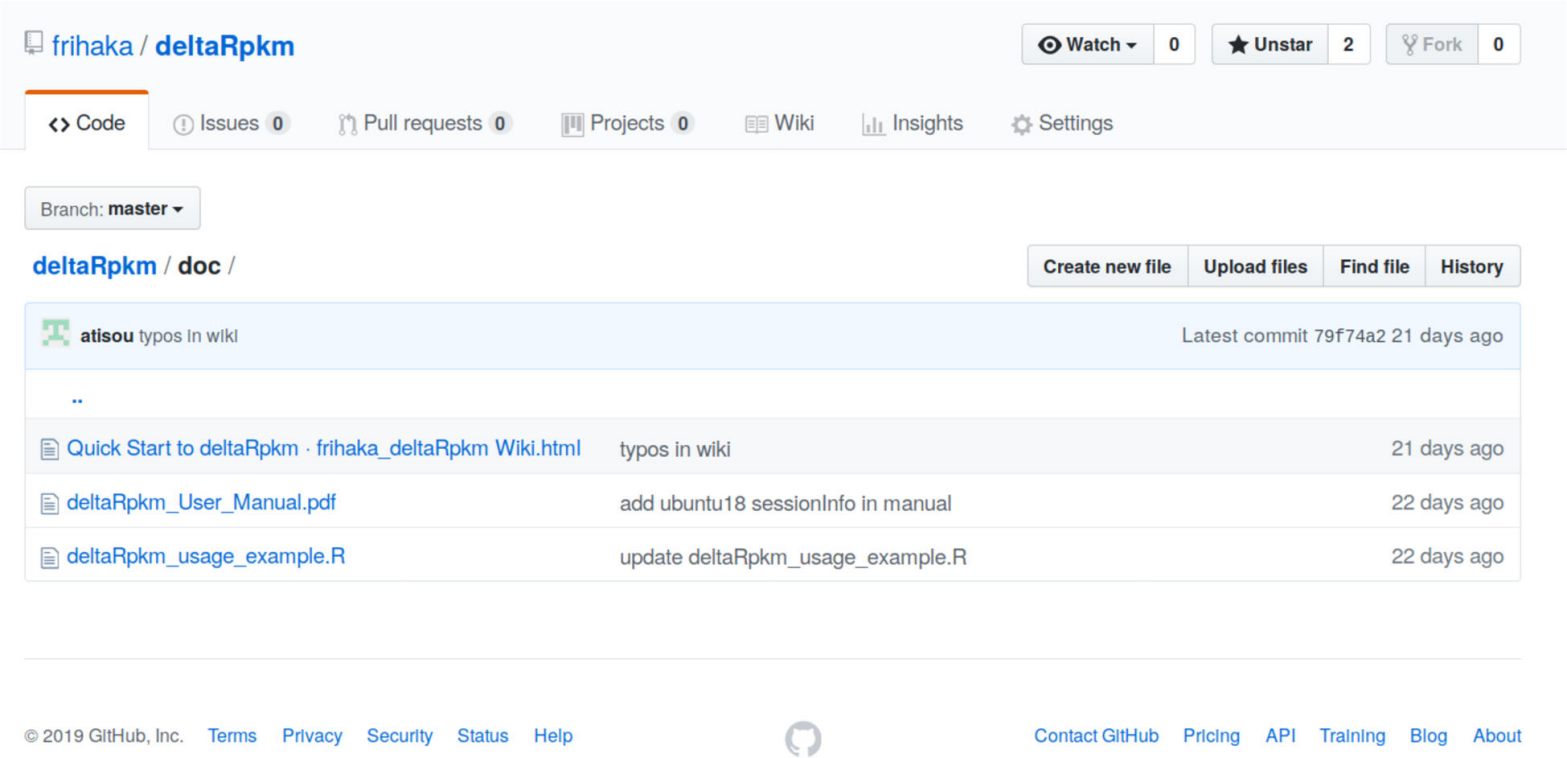
deltaRpkm on GitHub. Content of the documentation directory for full tutorials

The package provides working example datasets of different sizes from *Listeria monocytogenes* [1]. The complete documentation with more technical details, full tutorial and running R script can be downloaded from the deltaRpkm GitHub project (Fig. 4) and are also provided as Additional files 2 and 3.

## Results

The pipeline has been successfully applied in a recent publication [1] with *N* = 225 *Listeria monocytogenes* genomes annotated for their neurovirulence phenotype, as summarized in Fig. 3. Down-sampling tests show the robustness of the method (Additional file 1: Figure S2), with a consistent filtered gene set (Additional file 1: Figure S3). Analyzing a dataset of N = 225 samples takes less than 20 min (Additional file 1: Figure S4) while using less than 4GB of memory (Additional file 1: Figure S5), which makes deltaRpkm an ideal tool for desktop usage. Randomized genome groupings were performed as negative controls, giving shorter and non-robust lists of candidate genes (Additional file 1: Figure S6).

## Discussion

Our strategy in deltaRpkm has two main limitations: 1) the selection and use of a reference strain for read mapping, and consequently the detection of only differential presence of genes in that genome. But this could be overcome by using another strain for the mapping; 2) the non-detection of phenotypic core genes bearing mutations instead of being absent. Direct performance and feature comparisons with other tools are currently difficult, since deltaRpkm is the only one of its kind to perform comparative genomics bypassing the genome assembly and annotation steps. Nevertheless, the Table 2 summarizes the main features of deltaRpkm in comparison to two other nearest tools, BPGA [7] and Roary [6].

**Table 2:** Runtimes of deltaRpkm pipeline, versus two most similar tools. Since deltaRpkm does not require any assembly and annotation steps, it is difficult to compare it with other methods

Method	Small dataset	Large dataset
deltaRpkm	*N* = 31, runtime = ~ 40 s *(L.monocytogenes*, ~ 3 Mb*)*	N = 225, runtime = ~ 20 min *(L.monocytogenes)*
Roary [6]	N = 24, runtime = ~ 6 min (*S.enterica*, ~ 4.8 Mb) [6]	*N* = 1000, runtime = ~ 250 min (*S.enterica serovar Typhi*) [6]
BPGA [7]	*N* = 28, runtime = ~ 3 min (*S.pyogenes*, ~ 1.8 Mb) [7]	N = 1000, runtime = ~ 420 min (*S.pyogenes*) [7]

A powerful feature of deltaRpkm is the inclusion of non-coding genes in contrast to the classical pan-core-genome methods that only target protein-coding genes [4, 6, 7]. The whole genome of the reference is used, and even short non-coding elements are taken into account.

## Conclusions

deltaRpkm is a user-friendly R package that makes use of a standard gene counts table to infer a subset of genes potentially involved in a phenotype. The simplicity of its usage, combined with its scalability to large groups of whole genome datasets are the key features of deltaRpkm in the field of comparative genomics.

## Availability and requirements

Project name: deltaRpkm.

Project home page: https://github.com/frihaka/deltaRpkm

Operating system(s): Linux, MacOSX, Windows.

Programming language: R.

License: AGPL v3.

## Supplementary information

**Additional file 1: Figure S1.** RPKM values distribution of all genes in the dataset. This can be used to fine tune the heatmap color break parameters. **Figure S2.** Dataset size effect on the distribution of the δRPKM values. A. Boxplots for datasets from *N* = 7 to *N* = 225 samples. The dataset size does not influence the median δRPKM values that are used when computing the differentially present gene selection based on the 2*standard deviation of median δRPKM values. Two datasets are highlighted for illustration, *N* = 51 samples and *N* = 225 samples. B. Dataset size effect on threshold value (2*standard deviation) of median δRPKM. **Figure S3**. The selected differentially present gene set is robust. Downsampling shows that even with small size dataset, the identified genes highly overlap (*N* = 115) with the datasets of greater size**. Figure S4.** deltaRpkm performance: dataset size effect on runtime. The whole analysis pipeline with deltaRpkm can be run in less than 20 min in R for a dataset with N = 225 samples of *Listeria monocytogenes* (~ 3 Mb, ~ 3 K genes). Ubuntu 14.04, R 3.4.4, Intel Core i-4790 CPU @3.60Gzx8. **Figure S5.** deltaRpkm performance: dataset size effect on memory usage. The whole analysis pipeline with deltaRpkm uses less than 4G of memory in R for a dataset with N = 225 samples of *Listeria monocytogenes* (~ 3 Mb, ~ 3 K genes). Ubuntu 14.04, R 3.4.4, Intel Core i-4790 CPU @3.60Gzx8. **Figure S6.** deltaRpkm performance: real (A) versus randomized datasets (B). The gene differential presence gives shorter and non-robust list of genes when using randomized datasets of different sizes. Corrected RPKM.

**Additional file 2.** R usage script for a quick start.

**Additional file 3.** Complete documentation.

## Data Availability

The R package deltaRpkm standalone binaries for Linux, MacOS and Windows10 are available are https://github.com/frihaka/deltaRpkm, including tutorial and full documentation.

## Abbreviation

RPKM: Reads Per Kilobase per Million mapped reads
