# Supplementary material for: deltaRpkm: an R package for a rapid detection of differential gene presence between related bacterial genomes
Source: BMC Bioinformatics. 2019 Dec 2;20:621. doi: 10.1186/s12859-019-3234-2 (PMC6889214; doi:10.1186/s12859-019-3234-2)
Supplement: Supplementary file 1 — Additional file 1: Figure S1. RPKM values distribution of all genes in the dataset. This can be used to fine tune the heatmap color break parameters. Figure S2. Dataset size effect on the distribution of the δRPKM values. A. Boxplots for datasets from N = 7 to N = 225 samples. The dataset size does not influence the median δRPKM values that are used when computing the differentially present gene selection based on the 2*standard deviation of median δRPKM values. Two datasets are highlighted for illustration, N = 51 samples and N = 225 samples. B. Dataset size effect on threshold value (2*standard deviation) of median δRPKM. Figure S3. The selected differentially present gene set is robust. Downsampling shows that even with small size dataset, the identified genes highly overlap (N = 115) with the datasets of greater size. Figure S4. deltaRpkm performance: dataset size effect on runtime. The whole analysis pipeline with deltaRpkm can be run in less than 20 min in R for a dataset with N = 225 samples of Listeria monocytogenes (~ 3 Mb, ~ 3 K genes). Ubuntu 14.04, R 3.4.4, Intel Core i-4790 CPU @3.60Gzx8. Figure S5. deltaRpkm performance: dataset size effect on memory usage. The whole analysis pipeline with deltaRpkm uses less than 4G of memory in R for a dataset with N = 225 samples of Listeria monocytogenes (~ 3 Mb, ~ 3 K genes). Ubuntu 14.04, R 3.4.4, Intel Core i-4790 CPU @3.60Gzx8. Figure S6. deltaRpkm performance: real (A) versus randomized datasets (B). The gene differential presence gives shorter and non-robust list of genes when using randomized datasets of different sizes. Corrected RPKM. [file 12859_2019_3234_MOESM1_ESM.pdf]

Supplementary Information for

deltaRpkm: an R package for a rapid detection of differential gene presence between related genomes

Hatice Akarsu[1,4#], Lisandra Aguilar-Bultet[2,3,4#], Laurent Falquet[1,4*]

[1]Department of Biology, University of Fribourg, Switzerland,

[2]Institute of Veterinary Bacteriology, Vetsuisse Faculty, University of Bern, Bern, Switzerland,

[3]Graduate School for Cellular and Biomedical Sciences, University of Bern, Bern Switzerland,

[4]Swiss Institute of Bioinformatics, BUGFri group, Fribourg, Switzerland.

*To whom correspondence should be addressed.

[#] These authors contributed equally to this work.

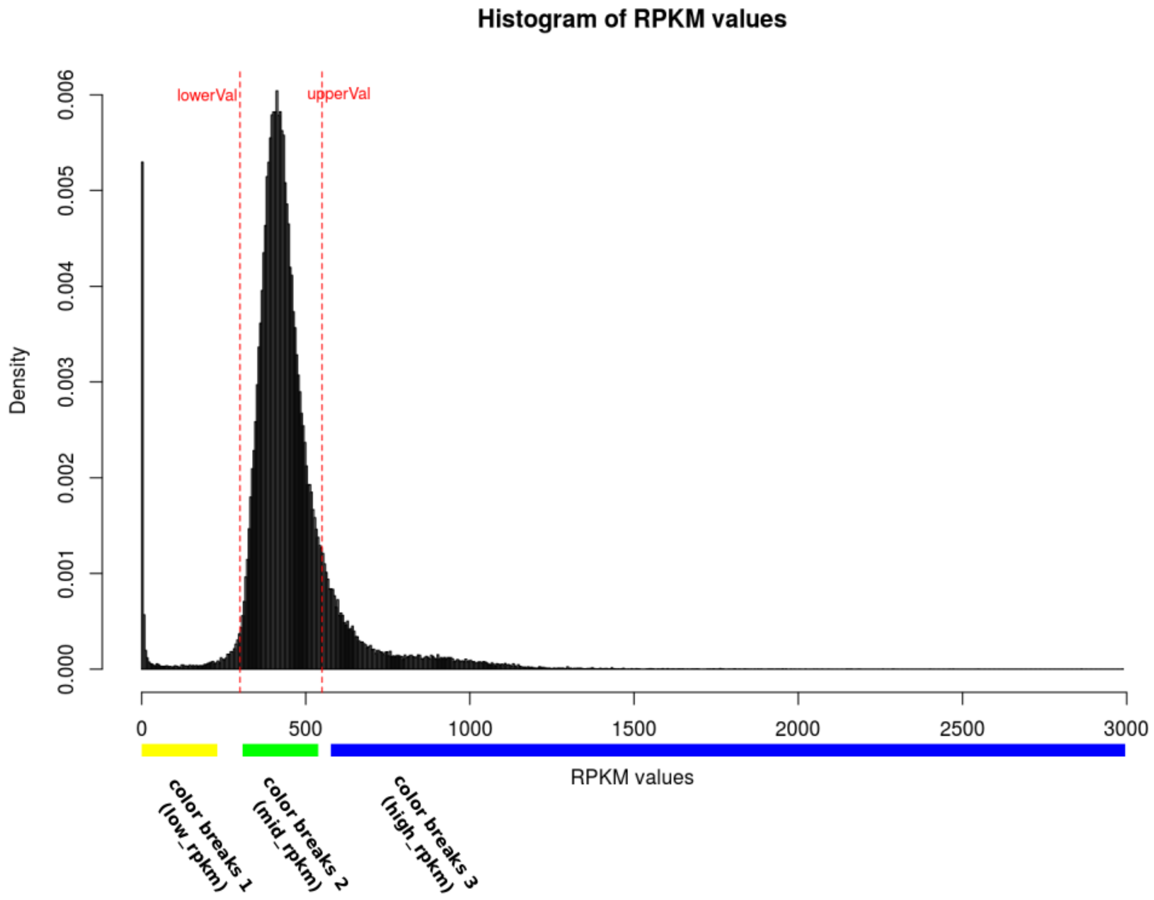

Fig. S1. RPKM values distribution of all genes in the dataset. This can be used to fine tune the heatmap colors breaks parameters.

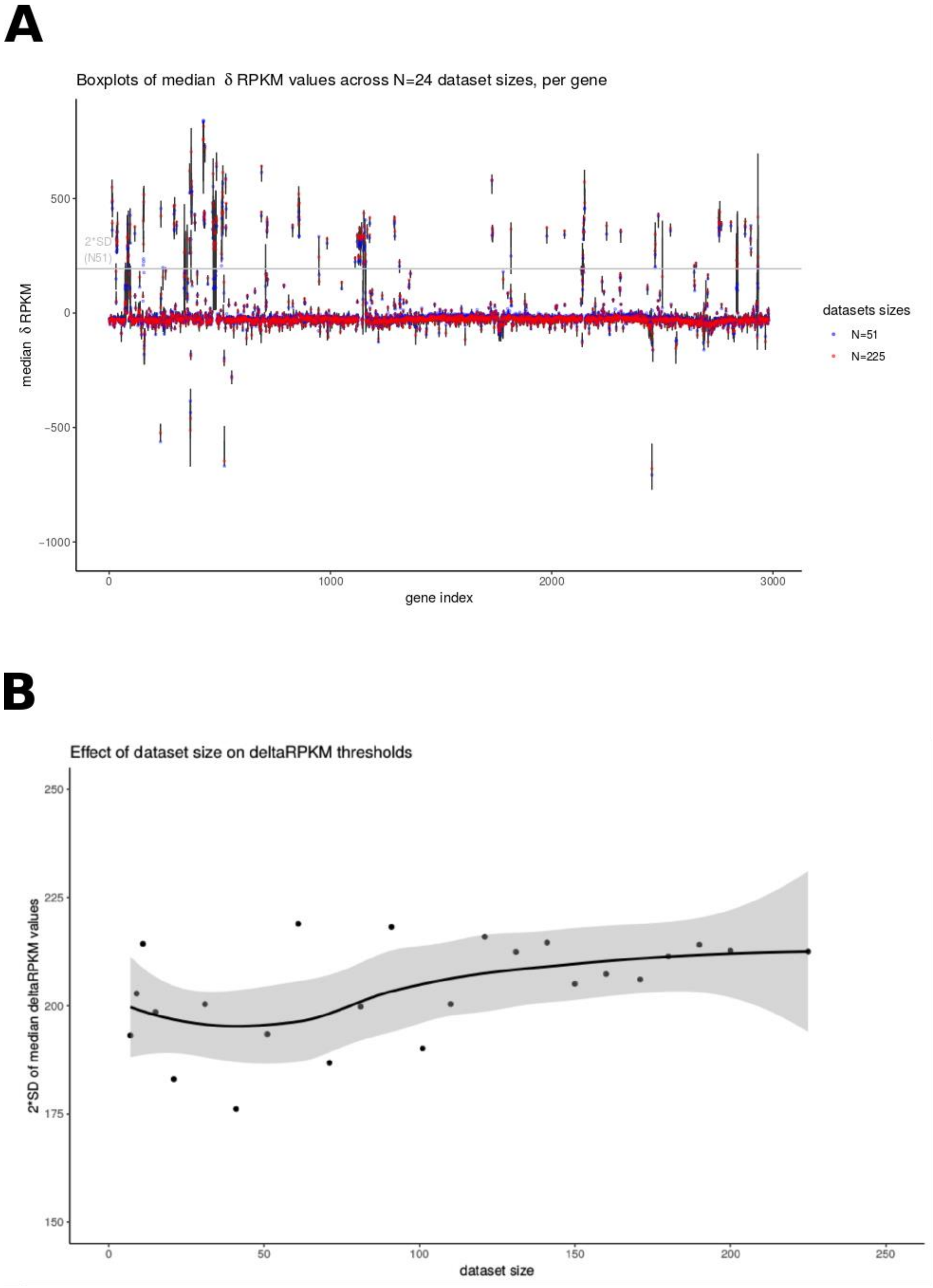

Fig. S2. Dataset size effect on *δRPKM* values distribution. A. Boxplots for datasets from N=7 to N=225 samples. The dataset size does not influence the median *δRPKM* values that are used when computing the differentially present gene selection based on the 2*standard deviation of median *δRPKM* values. Two datasets are highlighted for illustration, N=51 samples and N=225 samples. **B. Dataset size effect on threshold value (2*standard deviation) of median *δRPKM*.**

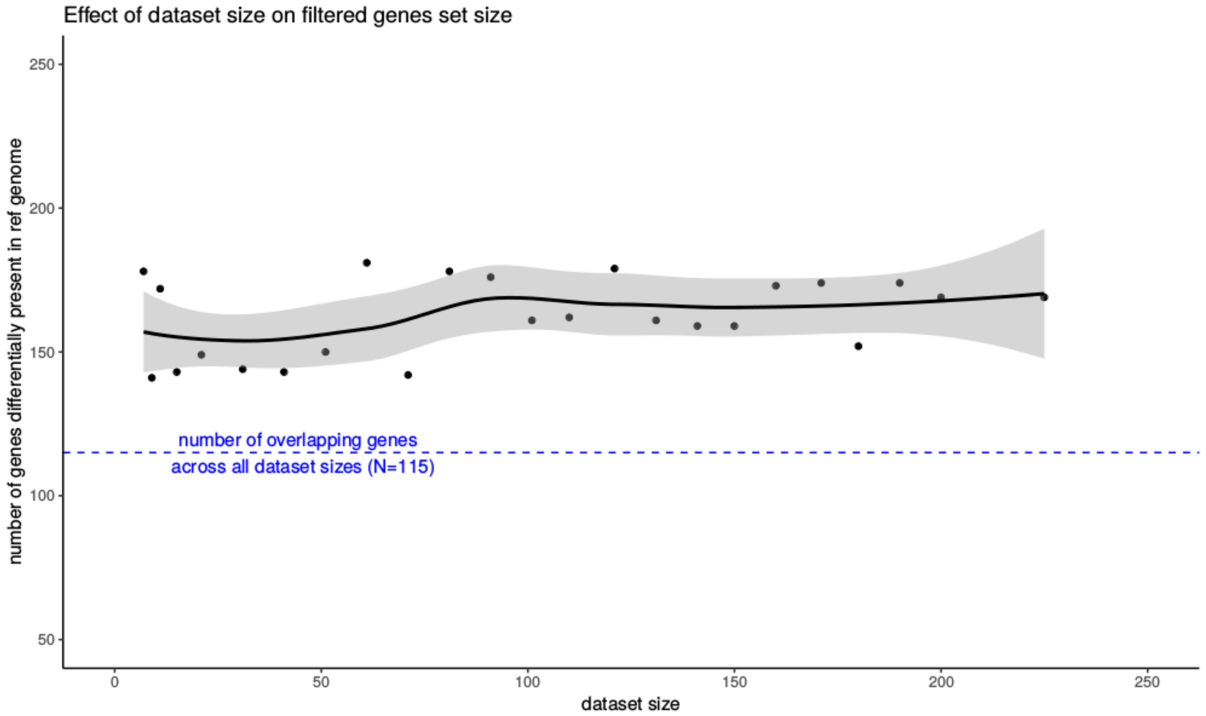

Fig. S3. The selected differentially present gene set is robust. Downsampling shows that even with small size dataset, the identified genes overlap (N=115) highly with the datasets of greater size.

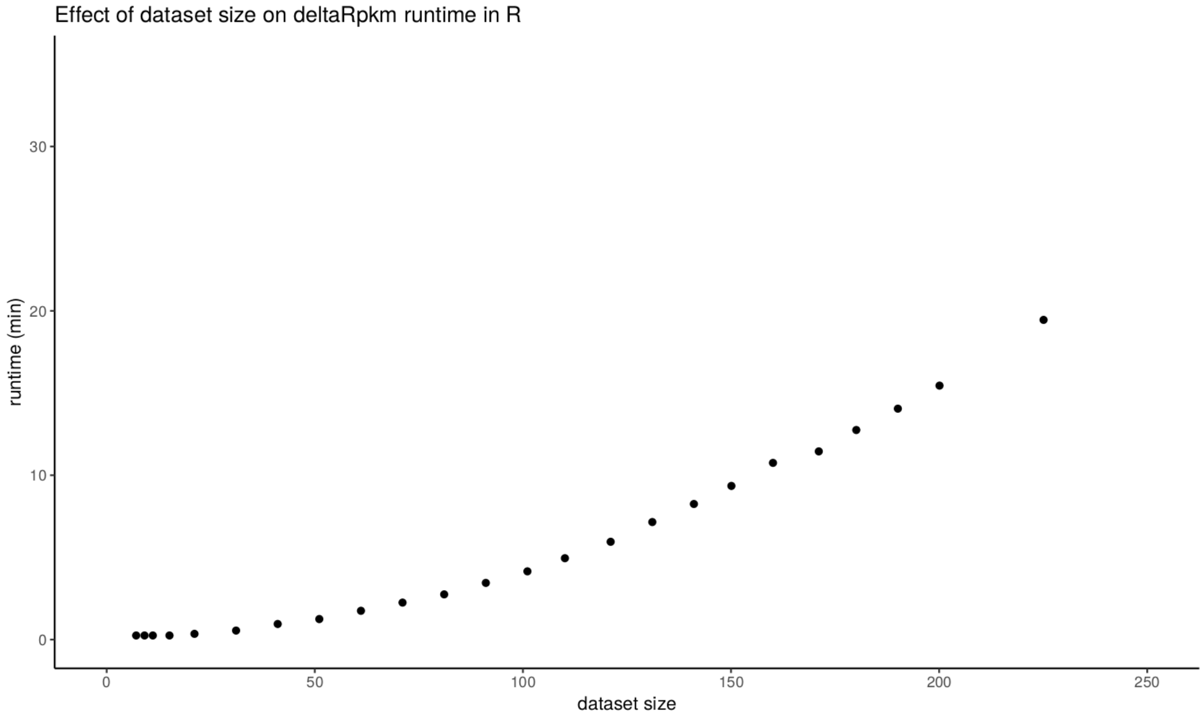

Fig. S4. deltaRpkm performance: dataset size effect on runtime. The whole analysis pipeline with deltaRpkm can be run in less than 20min in R for a dataset with N=225 samples of *Listeria monocytogenes* (~3Mb, ~3K genes). Ubuntu 14.04, R 3.4.4, Intel Core i-4790 CPU @3.60Gzx8.

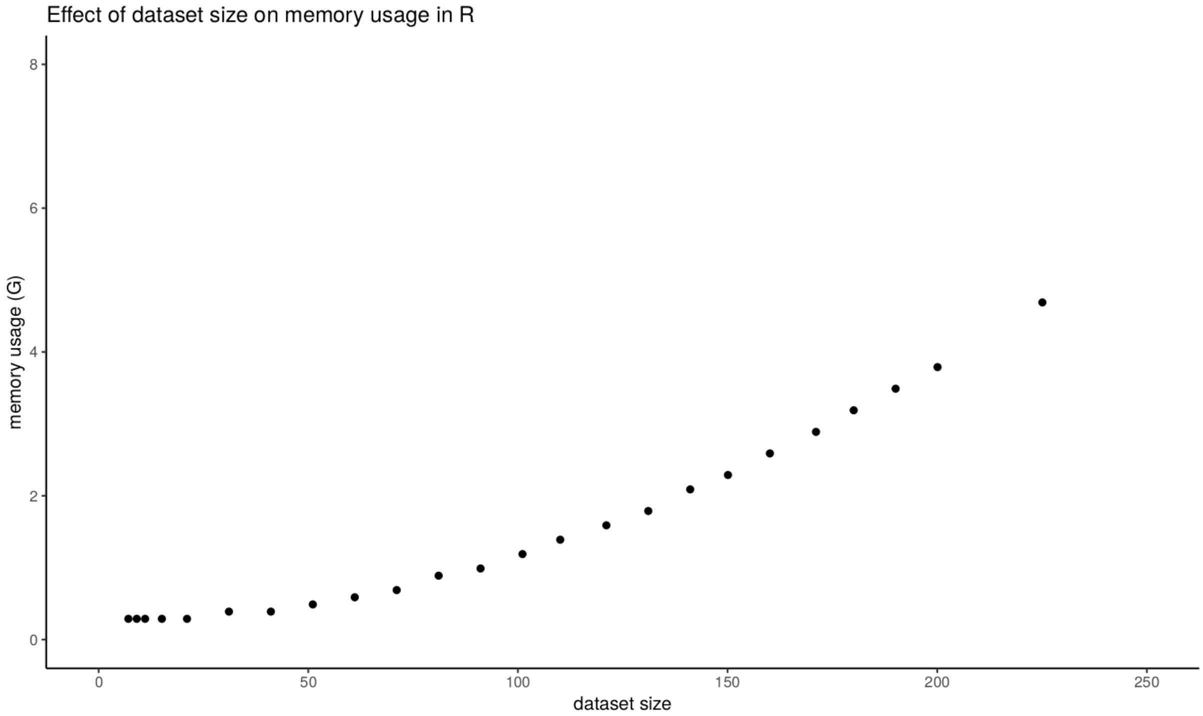

Fig. S5. deltaRpkm performance: dataset size effect on memory usage. The whole analysis pipeline with deltaRpkm uses less than 4G of memory in R for a dataset with N=225 samples of *Listeria monocytogenes* (~3Mb, ~3K genes). Ubuntu 14.04, R 3.4.4, Intel Core i-4790 CPU @3.60Gzx8.

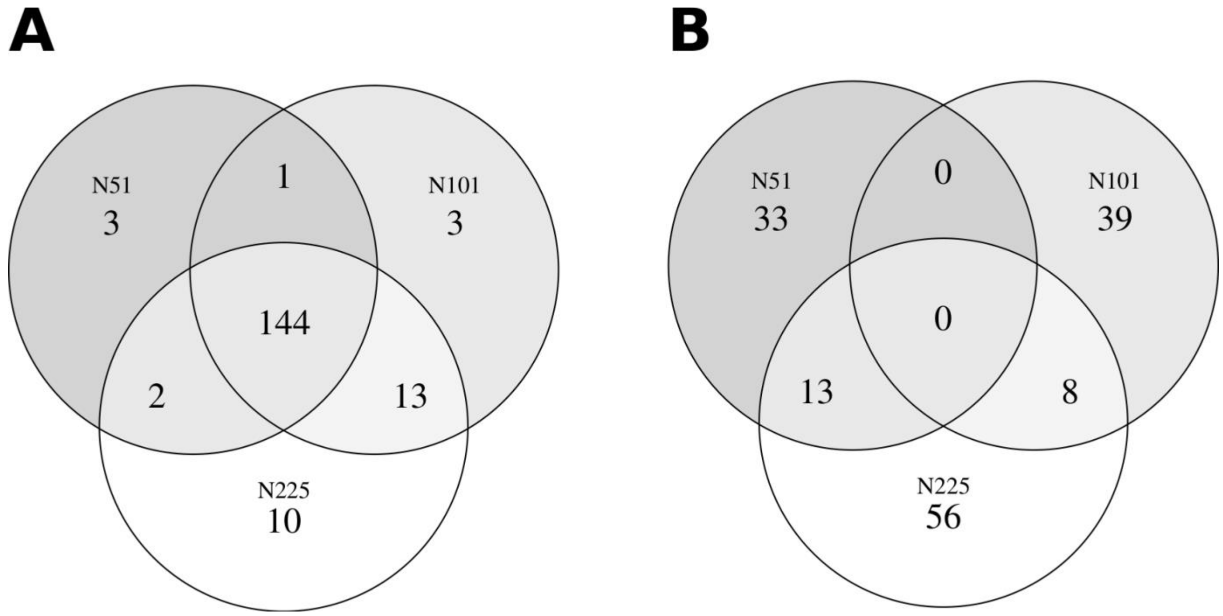

Fig. S6. deltaRpkm performance: real (A) versus randomized datasets (B). The genes differential presence gives shorter and non-robust list of genes when using randomized datasets of different sizes. Corrected RPKM.
