# Supplementary material for: deltaRpkm: an R package for a rapid detection of differential gene presence between related bacterial genomes
Source: BMC Bioinformatics. 2019 Dec 2;20:621. doi: 10.1186/s12859-019-3234-2 (PMC6889214; doi:10.1186/s12859-019-3234-2)
Supplement: Supplementary file 3 — Additional file 3. Complete documentation. [file 12859_2019_3234_MOESM3_ESM.pdf]

deltaRpkm

An R package for a rapid detection of differential gene presence between related genomes

Hatice Akarsu[1,2], Lisandra Aguilar-Bultet[3], Laurent Falquet[1,2]

[1]Department of Biology, University of Fribourg, Switzerland,

[2]Swiss Institute of Bioinformatics, BUGFri group, Fribourg, Switzerland

[3]Institute of Veterinary Bacteriology, University of Bern, Switzerland,

Nov 2019

deltaRpkm *is an R package whose main purpose is to quickly identify genes potentially involved in a trait of interest by performing a differential analysis of genes coverage between two sets of closely related bacterial genomes. The package provides functions to compute the RPKM, the* $\delta RPKM$*, candidate genes filtering and heatmap plot. It also includes methods to perform some batch effect controls and diagnostic plots.*

Contents

1 Prerequisites **3**
1.1 Input files . . . 3
1.2 Minimum of R knowledge . . . 3
1.3 R dependencies . . . 4
1.4 Download and install deltaRpkm . . . 4

2 Overview of the pipeline **5**

3 Building and loading the coverage table **5**

4 Loading the metadata table **6**

5 Convert read counts to RPKM values **8**
5.1 RPKM formula . . . 8
5.2 Run `deltaRpkm::rpkm` . . . 8

6 $\delta RPKM$ values **9**
6.1 $\delta RPKM$ formula . . . 9
6.2 Run `deltaRpkm::deltarpkm` . . . 9

7 Differential gene presence **9**
7.1 Strategy . . . 9
7.2 Run `deltaRpkm::deltaRPKMStats` . . . 10
7.3 Visual check of the m_j distribution . . . 11
7.4 Selected gene set . . . 11

8 Heatmap **12**
8.1 Rational . . . 12
8.2 Preparing the RPKM values for the heatmap . . . 12
8.3 Plot heatmap . . . 13
8.4 Tuning heatmap parameters: color breaks . . . 14

9 deltaRpkm performance: downsampling **16**
9.1 Dataset size effect on thresholding and gene set selection . . . 16
9.2 Dataset size effect on runtime . . . 18
9.3 Dataset size effect on memory usage . . . 19
9.4 Random datasets . . . 19

10 Binaries and OS platforms **20**
10.1 Ubuntu Trusty Tahr (14.04.5 LTS) . . . 20
10.2 Ubuntu Bionic Beaver (18.04.2 LTS) . . . 20
10.3 MacOS High Sierra (10.13.6) . . . 21
10.4 Windows10 . . . 22

1 Prerequisites

1.1 Input files

The deltaRpkm package requires 2 user input files:

1. a metadata table that provides parameter information for inter-group comparisons, with the following mandatory fields:

```
<sample> <phenotype_1> <phenotype_2>  <genome_length> <mapped_reads> <...>
```

   - **<sample>** the column containing the sample names
   - **<phenotype_1>** the column containing the name of the trait being investigated for the gene differential presence analysis
   - **<phenotype_2>** the column containing the 2nd trait that can be added in the heatmap for comparison
   - **<genome_length>** the column containing the reference genome length
   - **<mapped_reads>** the column containing the total number of mapped reads in each sample
   - **<...>**

   These are the minimum required elements to be given to the pipeline; more factors can be included for alternative analyses if desired.

2. a coverage table that combines the mapped read counts per gene and per sample, as this:

```
<chr> <start> <end> <geneID> <sample1_readCounts> <sample2_readCounts> <...>
```

   - **<chr>** the column containing the name of reference genome
   - **<start>** the column containing the gene start coordinate
   - **<end>** the column containing the gene end coordinate
   - **<geneID>** the column containing the gene identifier
   - **<sample1>** the column named by the sample 1 and containing its mapped read counts
   - **<sample2>** the column named by the sample 2 and containing its mapped read counts
   - **<...>**

Please make sure that the input tables follow as much as possible those formats (column order and names for the minimum required information). For instance, the sample names in the <sample> column of the metadata table MUST be the same as <sample_readCounts> in the coverage table.

The working examples provided by the package correspond to datasets of different sizes from *Listeria monocytogenes* (Aguilar-Bultet *et al.*, 2018).

1.2 Minimum of R knowledge

Although deltaRpkm is very simple/user-friendly and comes with an extensive documentation (`https://github.com/frihaka/deltaRpkm/tree/master/doc`), it assumes a minimum familiarity with R, *e.g* installing libraries from CRAN and Bioconductor, awareness of working environment, basic R commands and objects etc.

1.3 R dependencies

The R package deltaRpkm, like any R package, is built upon common R **CRAN** libraries that should be already installed in your system if you are an R user (ggplot2, ggfortify, dplyr, data.table...).

It also requires some **Bioconductor** R packages. If not already in your system, please make sure that the following R packages from Bioconductor - sva and Biostrings - are installed:

```
# for R >= 3.5
> if (!requireNamespace("BiocManager", quietly = TRUE))
    install.packages("BiocManager")
BiocManager::install("sva", version = "3.8")
BiocManager::install("Biostrings", version = "3.8")

# for R <= 3.4
source("https://bioconductor.org/biocLite.R")
biocLite("sva")
biocLite("Biostrings")
```

1.4 Download and install deltaRpkm

From GitHub https://github.com/frihaka/deltaRpkm/, download the compressed binary file on a local directory.

If you are familiar with R and have already most of the common R packages (ggplot2, ggfortify, dplyr...), you can install from the terminal with the `R CMD INSTALL` command:

```
R CMD INSTALL deltaRpkm_0.1.0_R_x86_64-pc-linux-gnu.tar.gz
```

and then just install the Bioconductor packages as shown above.
If you are not very familiar with R environment, try rather to install deltaRpkm with the function `install.packages()`, from inside R/RStudio:

```
> install.packages("path/2/deltaRpkm_0.1.0_R_x86_64-pc-linux-gnu.tar.gz",
    repos = NULL,
    dependencies = TRUE)
```

This will download your missing CRAN libraries that are required by deltaRpkm. You will still need to install the Bioconductor packages, as shown above.

2 Overview of the pipeline

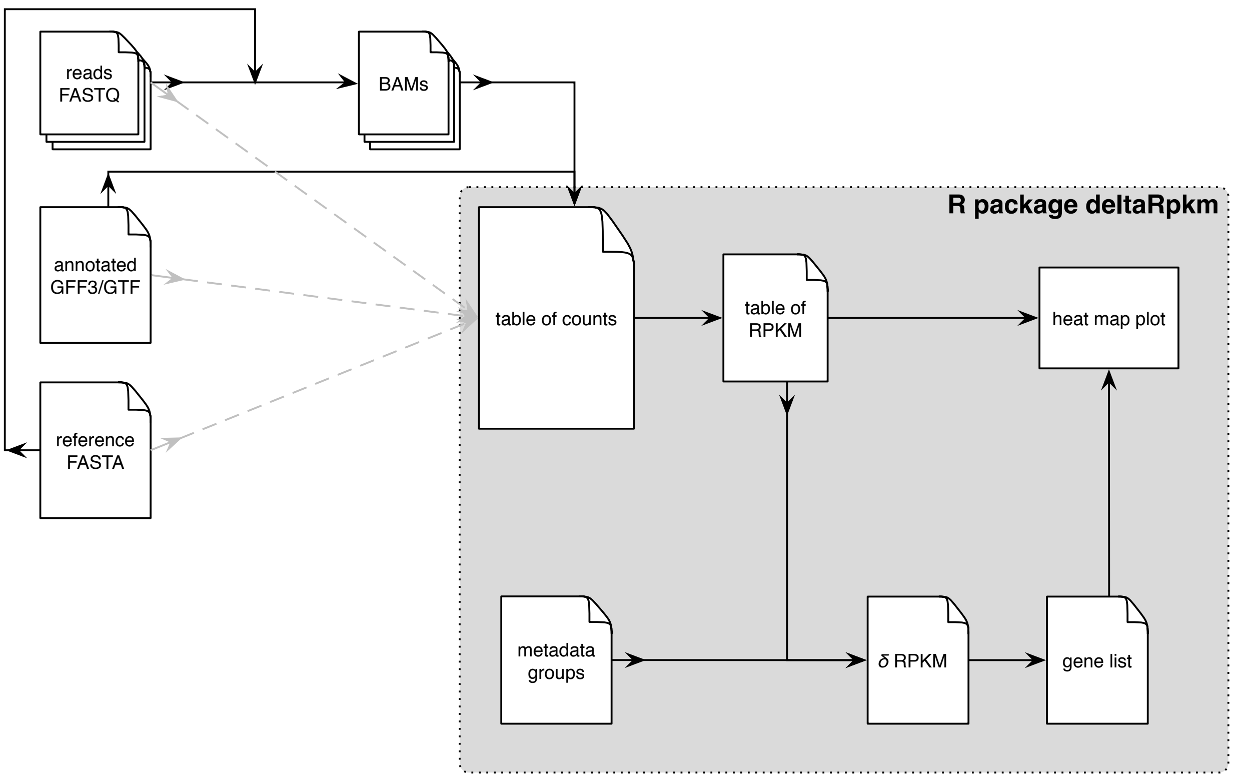

Figure 1: Overview of the deltaRpkm pipeline.

3 Building and loading the coverage table

The read counts per gene table must be pre-computed and provided by the user. Below is the command used to build it with `bedtools multicov` on a terminal:

```
bedtools multicov -bams aln.1.bam ... aln.n.bam -bed <bed/gff/vcf> > coverage_table.csv
```

Notes on `bedtools multicov`:

- any number of bam files can be run together in a batch mode, thus allowing all the samples of the dataset to be included in the coverage table
- do not forget to redirect/output the results into a coverage table

The user can build the read count table with other methods, like the RNA-seq aligner called STAR that maps and produces the coverage table at once.

Please do not forget to ensure that your custom coverage table (either produced by bedtools multicov or by STAR etc) follows the following required format:

- **<chr>**
- **<start>**
- **<end>**
- **<geneID>**
- **<sample1_readCounts>**
- **<sample2_readCounts>**
- <...>

Alternatively, example datasets derived from Aguilar-Bultet *et al.*, 2018 are available in the deltaRpkm package:

```
> data("coverage_table_N51")  # this creates coverage_table df in the
   environment
> head(coverage_table_N51[, 1:8])
                  chr start  end         geneID JF4931 JF5172 JF5761
     JF5827
1 JF5203_chromosome   318 1674 LMJF5203_00001   3109   1466   5582
   5761
2 JF5203_chromosome  1867 3013 LMJF5203_00002   2778   1099   4737
   4882
3 JF5203_chromosome  3120 4464 LMJF5203_00003   3218   1473   4914
   5365
4 JF5203_chromosome  4577 4865 LMJF5203_00004    947    358   1568
   1546
5 JF5203_chromosome  4868 5981 LMJF5203_00005   2578    932   4415
   4572
6 JF5203_chromosome  6029 7970 LMJF5203_00006   4125   1853   7018
   7681
```

4 Loading the metadata table

The user must provide a metadata table with some minimum informations/columns about the samples:

- a column containing the trait or characteristic 1 data, that will be used for the RPKM comparisons. This is the main characteristic of interest being studied, *i.e* the trait of group 1 with the reference genome. This trait is the criteria of categorizing the datasets into 2 distinct groups and the basis of the whole comparison. For example: "lineage_type" which can include different values but only 2 will be compared. That is why we advise to categorize samples into groups; in the example: Lineage_I and Lineage_II
- a column containing the trait 2 data, that will be used as a colored sidebar of the heatmap: this corresponds to a 2nd characteristic that the user can add to check whether the clustering in the heatmap correlates with this 2nd trait. For example: "infection_origin"

- reference genome length
- total number of mapped reads

The metadata/design table must be a data frame that looks like this:

Table 1: Example of input metadata table.

sample	platform	lineage_type	infection	genome_length	mapped_reads
JF4839	HiSeq2000	Lineage_II	ENV	2900890	8288011
JF4899	HiSeq4000	Lineage_I	CNS	2900890	9797440
JF4901	HiSeq2000	Lineage_I	CNS	2900890	1926369
JF4902	HiSeq2000	Lineage_I	CNS	2900890	1750981
JF4904	HiSeq2000	Lineage_I	CNS	2900890	1469430

A working example metadata dataset from deltaRpkm package is shown below:

```
> data("metadata_table_N51")  # this creates metadata_table df in the
   env
> head(metadata_table_N51)
  sample   platform  lineage_type  infection  genome_length  mapped_reads
1  JF4906  HiSeq2000     Lineage_I        CNS        2900890       2042865
2  JF4929  HiSeq4000     Lineage_I        CNS        2900890       9469100
3  JF4931  HiSeq3000    Lineage_II        CNS        2900890       5285534
```

Format the metadata information with `deltaRpkm::loadMetadata` function, giving in as arguments:

- **user_metadata** = <data frame of user input design table>
- **delta_phenotype_colname** = <phenotype 1 column name used to build the 2 categories and perform the comparison>
- **heatmapbar_phenotype_colname** = <phenotype 2 column name used to build the extra bar in the heatmap>
- **samples_colname** = <column name containing the sample IDs>
- **genome_length_colname** = <genomic length (in bp) of the reference genome used for mapping>
- **mapped_reads_colname** = <total number of mapped reads, for each sample ID>

```
> design_table <- loadMetadata(user_metadata = metadata_table_N51,
                               delta_phenotype_colname = "lineage_type
                                  ",
                               heatmapbar_phenotype_colname = "
                                  infection",
                               samples_colname = "sample",
```

```
                                  genome_length_colname = "genome_length"
                                      ,
                                  mapped_reads_colname = "mapped_reads")

> head(design_table)
  sample lineage_type infection genome_length mapped_reads
1 JF4906     Lineage_I       CNS       2900890      2042865
2 JF4929     Lineage_I       CNS       2900890      9469100
3 JF4931    Lineage_II       CNS       2900890      5285534
```

5 Convert read counts to RPKM values

5.1 RPKM formula

deltaRpkm uses the **Reads Per Kilobase Million RPKM** - a standard RNA-seq metrics that normalizes the read counts per gene for **Sequencing Depth** and **Gene Length**:

with N_s being the total number of read counts in the sample,

$$scalingFactor = \frac{N_s}{10^6} \quad (1)$$

$$RPM = \frac{readCountPerGene}{scalingFactor} \quad (2)$$

$$RPKM = \frac{RPM}{geneLength.10^{-3}} \quad (3)$$

The equation (2) corresponds to the normalization of the read counts by the sample sequencing depth; and equation (3) corresponds to the normalization by the gene length.

5.2 Run **deltaRpkm::rpkm**

Run the following `deltaRpkm::rpkm` function to compute the RPKM values of each gene, in each sample:

```
> rpkmtable <- rpkm(user_metadata = design_table,
                    coverage_table = coverage_table_N51,
                    delta_phenotype_colname = "lineage_type",
                    heatmapbar_phenotype_colname = "infection")
> head(rpkmtable)
  sample          geneID lineage_type infection reads rpkm
1 JF4906 LMJF5203_00001    Lineage_I       CNS  1177  425
2 JF4906 LMJF5203_00002    Lineage_I       CNS   952  406
3 JF4906 LMJF5203_00003    Lineage_I       CNS  1080  393
```

6 $\delta RPKM$ values

6.1 $\delta RPKM$ formula

The analysis is centered around a pairwise comparison of gene presence/absence between genomes categorized into two different groups following the selected trait or feature:

- a group 1 that shares the trait A of the reference genome
- a group 2 that does not have the reference trait A

For each pairwise comparison of a gene j between a genome x from group 1 and a genome y from group 2, `deltaRpkm::deltarpkm` function computes the difference of their RPKM values at gene j ($\delta RPKM_{j_{xy}}$) as:

$$\delta RPKM_{j_{xy}} = RPKM_{j_x} - RPKM_{j_y} \quad (4)$$

6.2 Run **deltaRpkm::deltarpkm**

```
> deltarpkm_table <- deltarpkm(rpkm_table = rpkmtable,
                               genes_names = unique(rpkmtable$geneID),
                               samples_colname = "sample",
                               delta_phenotype_colname = "lineage_type
                                  ",
                               reference_sample = "JF5203",
                               nonref_delta_phenotype = "Lineage_II")
> head(deltarpkm_table)
 geneID sample.group1 lineage_type.group1 infection.group1 reads.group1
      rpkm.group1 sample.group2 lineage_type.group2 infection.group2
     reads.group2 rpkm.group2 deltarpkm
1 LMJF5203_00001         JF4906          Lineage_I              CNS
                    1177          425          JF4931           Lineage_II
                 CNS                        3109          433         -8
2 LMJF5203_00001         JF4906          Lineage_I              CNS
                    1177          425          JF5172           Lineage_II
                 CNS                        1466          520        -95
3 LMJF5203_00001         JF4906          Lineage_I              CNS
                    1177          425          JF5761           Lineage_II
                 ENV                        5582          465        -40
```

This run might take a few minutes, depending on the size of the datasets.

7 Differential gene presence

7.1 Strategy

The deltaRpkm package main feature is to screen for *the preferential presence of genes in the reference genome group, versus a comparison group.*
We use the method `deltaRpkm::deltaRPKMStats` to infer this set of genes, since they could potentially be involved in the reference genome group trait (<lineage_type> =”Lineage_type_I”). This function:

1. computes for each gene j the **median value of all its $\delta RPKM$ (m_j)** derived from the sample pairwise comparisons. Note: a negative median value of all $\delta RPKM$ of a given gene would mean that this gene is "preferentially present" in the comparison samples of group 2 than in the reference genome group 1

2. calculates the **standard deviation** s of all the m_j values in the analysis

3. selects genes as present in the reference genome group 1 based on an **arbitrary threshold of** $2.s$:

$$selectedGene : m_j >= 2.s \tag{5}$$

   In other words, a gene j having a median $\delta RPKM$ value greater than $2.s$ will be considered as "preferentially present" in the reference genome group 1 (with <lineage_type> ="Lineage_type_I") than in the comparison group 2 (with <lineage_type> ="Lineage_type_II").

7.2 Run `deltaRpkm::deltaRPKMStats`

```
> stats_table <- deltaRPKMStats(deltarpkm_table = deltarpkm_table)
> head(stats_table)
          geneID sample.group1 lineage_type.group1 infection.group1
             reads.group1 rpkm.group1 sample.group2 lineage_type.
             group2
1 LMJF5203_00001        JF4906           Lineage_I              CNS
          1177          425        JF4931          Lineage_II
2 LMJF5203_00001        JF4906           Lineage_I              CNS
          1177          425        JF5172          Lineage_II
3 LMJF5203_00001        JF4906           Lineage_I              CNS
          1177          425        JF5761          Lineage_II
  infection.group2 reads.group2 rpkm.group2 deltarpkm deltarpkm_median
      deltarpkm_medianSD thres_SD_median_value selected_gene
1              CNS          3109         433        -8              -31
             114.24                   228.48              -
2              CNS          1466         520       -95              -31
             114.24                   228.48              -
3              ENV          5582         465       -40              -31
             114.24                   228.48              -
```

The default threshold value to select genes is based on $2.s$. But this threshold can be changed in the `deltaRpkm::deltaRPKMStats` parameter `min_SD_foldChange`, *e.g*:

```
> stats_table_fcnew <- deltaRPKMStats(min_SD_foldChange = 1.5,
                                      deltarpkm_table = deltarpkm_table)
```

Note that the column **`selected_gene`** contains information about whether a given gene should be selected as present preferentially in the reference genome group (noted as "+") or not (noted as "-"). This column will be used later to filter the relevant genes.

7.3 Visual check of the m_j distribution

With the function deltaRpkm::median_plot it is possible to check visually the median $\delta RPKM$ value of every gene (one dot per gene).

```
> median_plot(data_table = stats_table,
              gene_annotation_table = coverage_table_N51) # genes
                annotation info
```

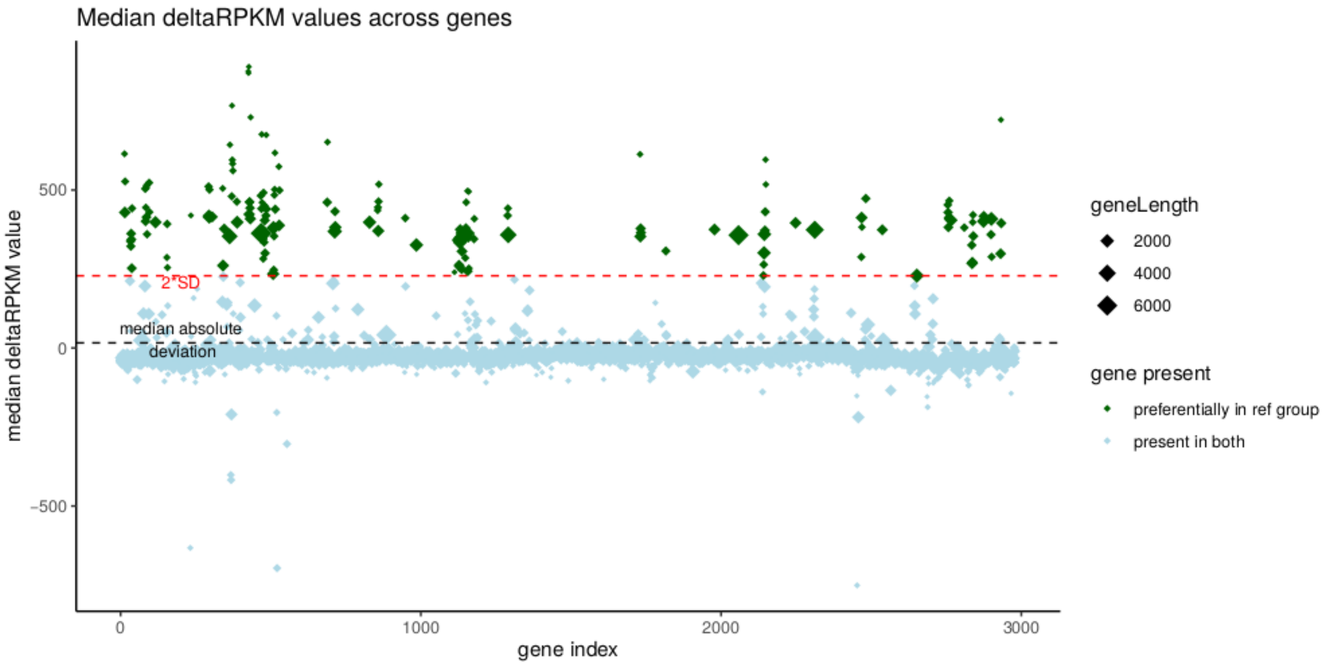

Figure 2: Median $\delta RPKM$ values for all genes. Plot output from deltaRpkm::median_plot.The negative median $\delta RPKM$ values correspond to genes that appear as better covered in the comparison group 2 than in the reference genome group 1. Note: the gene index value reflects the genomic coordinates since they are ordered as the gene names (these later being themselves given during *de novo* annotation based on the genomic coordinates, roughly speaking).

The genes in dark green in **Figure 2** correspond to the set of genes present in the reference genome group 1 and potentially linked to the trait of interest ("lineage_type").

7.4 Selected gene set

For a given threshold value of median $\delta RPKM$ (by default $2.s$ of all median $\delta RPKM$ values), we can simply extract the genes appearing as differentially present in the reference genome group 1 (green dots in the **Figure 2**):

```
> differential_present_genes <- unique(stats_table[stats_table$
   selected_gene %in% "+", ]$geneID)
> length(differential_present_genes)
> [1] 173
> head(differential_present_genes)
> [1] "LMJF5203_00013" "LMJF5203_00014" "LMJF5203_00015" "LMJF5203_
```

```
    00033" "LMJF5203_00034" "LMJF5203_00035"
```

This gene set can be used to perform various functional clustering analysis. We propose in the package a method to build a summary heatmap of their RPKM values and how they relate (or not) to a second trait of interest.

8 Heatmap

8.1 Rational

The idea is to analyse how the RPKM values of the genes specific to the reference genome group 1 distribute across all samples of group 1 and group 2. The aim is:

- to confirm (or infirm) that the heatmap clustering of the samples into two distinct categories is coherent with the initial group 1 and group 2 definition. Typically, the selected genes should present in overall higher RPKM values in the reference genome group 1 than in group 2. But it is not always the case.
- to investigate at a higher resolution the homogeneity of each group

Thus deltaRpkm allows to investigate the clustering of the selected genes based on their RPKM values computed earlier. A putative correlation with the second trait can be visualized by adding a color bar corresponding to this second trait given in the metadata table - which is "infection" in the working example dataset.
The heatmap plot is made with the `deltaRpkm::rpkmHeatmap` function, derived from the `gplots::heatmap.2` method (Warnes et al., 2018).

8.2 Preparing the RPKM values for the heatmap

The heatmap will focus only on the *RPKM values of the set of genes that are relevant*, *i.e* the ones that appear as differentially present in the reference genome group 1 (see the darkgreen dots in **Figure 2**).

For this, we first subset the RPKM data table and keep only the rows/genes that were selected using the `deltaRpkm::subsetRPKMTable`:

```
# Subset the RPKM table for the selected genes
> heatmap_table <- subsetRPKMTable(rpkm_table = rpkmtable,
                          user_metadata = design_table,
                          delta_phenotype_colname = "lineage_type",
                          heatmapbar_phenotype_colname = "infection",
                          sd_filtered_genes = differential_present_genes
                             )
> head(heatmap_table)
  sample lineage_type infection LMJF5203_00013 LMJF5203_00014 LMJF5203
     _00015 LMJF5203_00033 LMJF5203_00034 LMJF5203_00035 LMJF5203_
     00036
1 JF4906     Lineage_I        CNS             607             450
                630            421            445            521
                553
```

```
 2  JF4929      Lineage_I         CNS              581              397
                  498              456              447              427
                  423
 3  JF4931     Lineage_II         CNS                0                0
                    0               68                1                2
                    1
```

Then the subsetted RPKM values data frame is converted to a matrix (since this is the required format for the heatmap function) using the `deltaRpkm::convertHeatmapToMatrix` function:

```
# Convert the subsetted RPKM table to a matrix
> heatmap_matrix <- convertHeatmapToMatrix(wide_rpkm_table = heatmap_
    table,
                                    delta_phenotype_colname = "lineage_type",
                                    heatmapbar_phenotype_colname = "infection"
                                      )
> head(heatmap_matrix)
       LMJF5203_00013 LMJF5203_00014 LMJF5203_00015 LMJF5203_00033
          LMJF5203_00034 LMJF5203_00035 LMJF5203_00036 LMJF5203_00037
          LMJF5203_00038 LMJF5203_00082 LMJF5203_00083 LMJF5203_00084
JF4906              607            450            630            421
              445            521            553            472
              491            619            352            450
JF4929              581            397            498            456
              447            427            423            412
              484              0              0              0
JF4931                0              0              0             68
                1              2              1              0
                1              0             18            457
```

It is important to note that the heatmap matrix must contain sample names as row names.

8.3 Plot heatmap

Finally, we create a summary plot as a heatmap to highlight difference in the RPKM values of the selected genes between samples of group 1 and group 2, using the `deltaRpkm::rpkmHeatmap` function:

```
> rpkmHeatmap(filtered_rpkm_matrix = heatmap_matrix,
              user_metadata = design_table,
              heatmapbar_phenotype_colname = "infection")
```

This creates an output heatmap file `deltaRpkm_heatmap.tiff` in the working directory. The heatmap for the example dataset (*Listeria monocytogenes, N=51*) is shown in **Figure 3**. It confirms the clustering of the samples into the initial two categories: group 1 samples cluster together on the upper part corresponding to high RPKM values, while group 2 samples cluster together in the lower part of the heatmap with lower RPKM values, for the selected gene set. The heatmap colors can be easily changed with the color break parameters:

```
> rpkmHeatmap(filtered_rpkm_matrix = heatmap_matrix,
              user_metadata = design_table,
              heatmapbar_phenotype_colname = "infection",
              low_color = "col1",  # low RPKM values, default "yellow"
              mid_color = "col2",  # mid range RPKM values, default "
                 green"
              high_color = "col3") # high range RPKM values, default "
                 blue"
```

See the next section for more on color breaks tuning.

8.4 Tuning heatmap parameters: color breaks

`deltaRpkm::rpkmHeatmap` comes with various parameters options (see `?rpkmHeatmap`), some derived from the original `gplots::heatmap.2`, and some specific to deltaRpkm analysis.
In particular, the heatmap **color breaks** can be adjusted with i) the `binsize` (default 200), ii) the `lower_limit` (default value 300) and iii) `upper_limit` (default value 550) arguments. These values are based on the distribution of the RPKM values and correspond to the lower and upper boundary RPKM values of the main peak:

```
> hist(rpkmtable$rpkm, freq = FALSE, breaks = 1000)
```

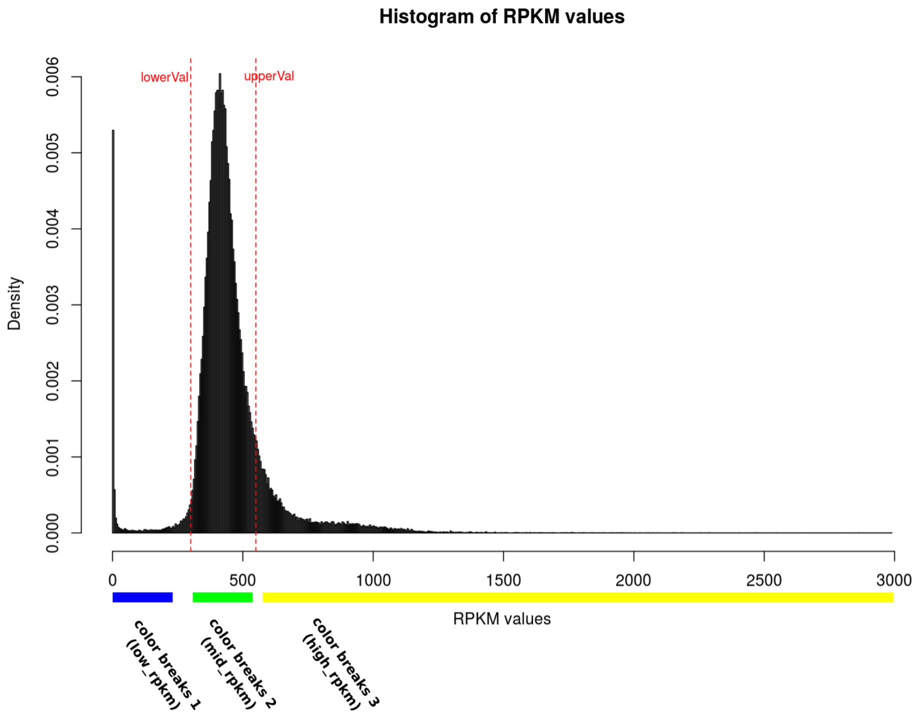

Figure 4: Distribution of RPKM values: inferring the heatmap color breaks from the histogram main peak boundary values. The lower (~300) and upper (~550) values of RPKM are used in the `deltaRpkm::rpkmHeatmap` to adjust the heatmap color breaks. Working dataset *Listeria monocytogenes, N=51.*

Also, deltaRPKM proposes some methods to infer these RPKM boundary values with the function `deltaRpkm::boundaries`:

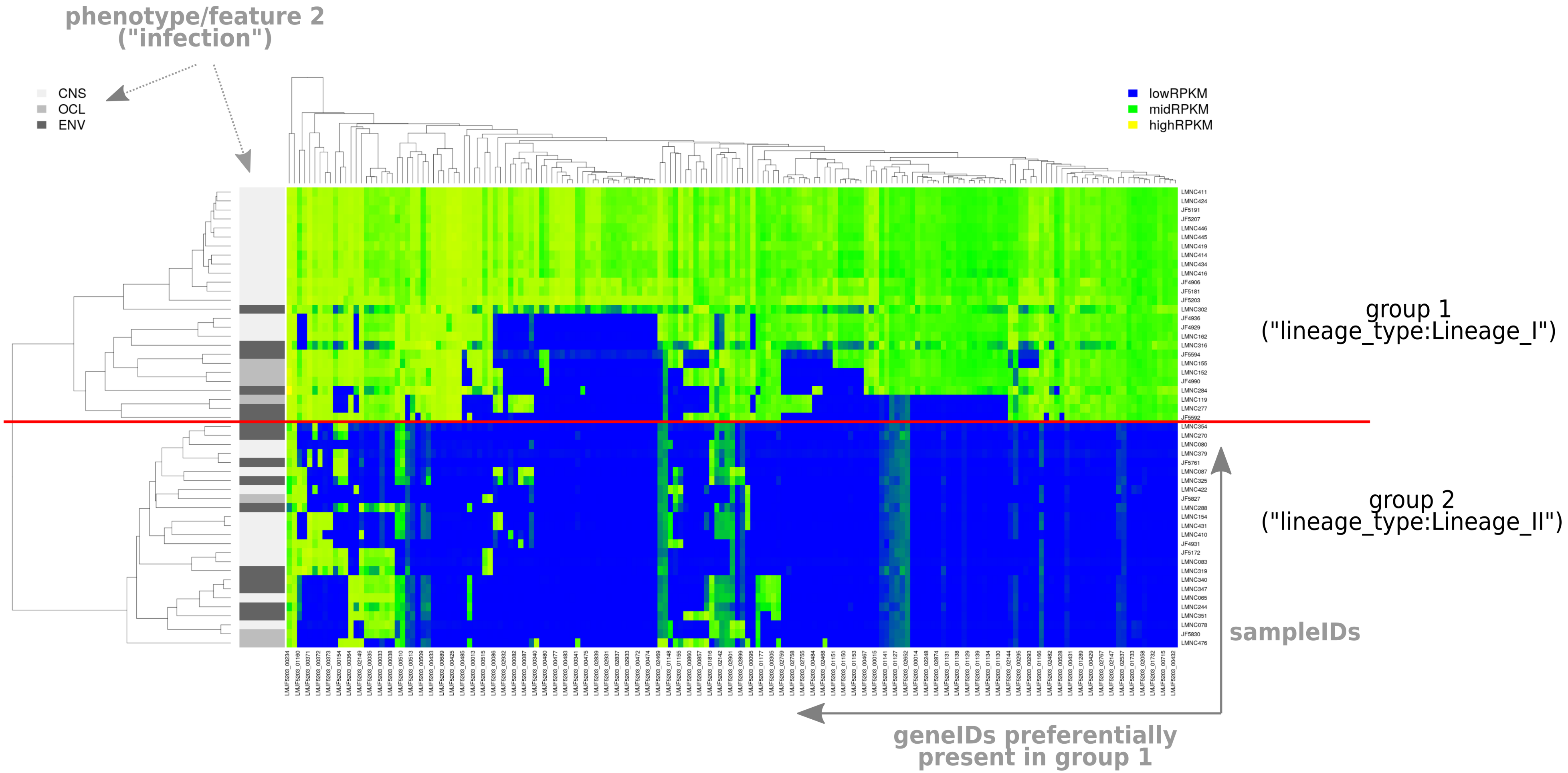

Figure 3: RPKM values distribution of the selected across samples from group 1 and group 2. Plot output from `deltaRpkm::rpkmHeatmap`. The samples cluster following the trait 1 ("lineage_type"), with group 1 annotated as "Lineage_I" and group 2 annotated as "Lineage_II". Most of the selected genes appear with a low RPKM value in group 2 cluster, even though some group 2 samples present high RPKM values (blue pixels) for certain geneIDs, suggesting these genes as potential false positives.

```
# default method mclust
> res <- boundaries(x = rpkmtable$rpkm)
> res$boundaries_df
  boundaries rpkm_values
1 lower_limit         300
2 upper_limit         624

> res <- boundaries(x = rpkmtable$rpkm, strategy = "ratios")
>    boundaries rpkm_values
1 lower_limit         295
2 upper_limit         585

> res <- boundaries(x = rpkmtable$rpkm, strategy = "quartiles")
>    boundaries rpkm_values
  boundaries rpkm_values
1 lower_limit         383
2 upper_limit         487
```

`deltaRpkm::boundaries` applies by default the **`mclust`** parameter, which is derived from the method `mclust::densityMclust`. This can be changed with the parameter strategy. The boundary RPKM values can be simply extracted as `res$boundaries_df` containing the RPKM boundary values of interest.

Feel free to play with these RPKM boundary and color break parameter values in rpkmHeatmap function and observe the effect(s) on the heatmap readout.

9 deltaRpkm performance: downsampling

The initial *Listeria monocytogenes* dataset of N=225 samples is downsampled up to N=7 samples. Each dataset is run through deltaRpkm pipeline and the different outcomes are compared.

9.1 Dataset size effect on thresholding and gene set selection

The gene differential presence is based on a threshold value defined as 2 times (default value) the standard deviation of the medians of $\delta RPKM$ values. The median plots (**Figure 2**) for all the datasets of different sizes can be summarized in a single boxplot per gene, as shown in **Figure 5**.

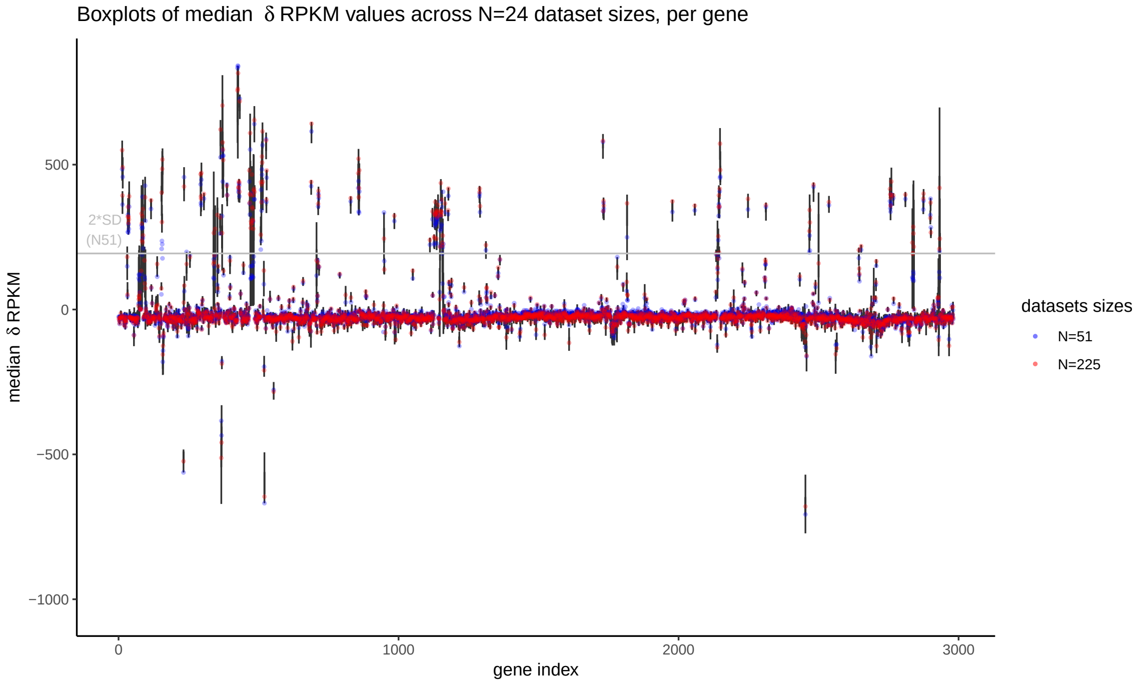

Figure 5: Dataset size effect on the median $\delta RPKM$ distribution (boxplots series). 24 datasets of different sizes (N=225 to N=7 samples) are plotted as boxplots, one per gene. Datasets with N=51 and N=225 samples are highlighted to evaluate the degree of $\delta RPKM$ variation between datasets of different sizes.

The boxplot series of **Figure 5** shows that most of the selected genes present a stable median $\delta RPKM$ distribution across all dataset sizes (bars fully above the $2.s$ threshold).

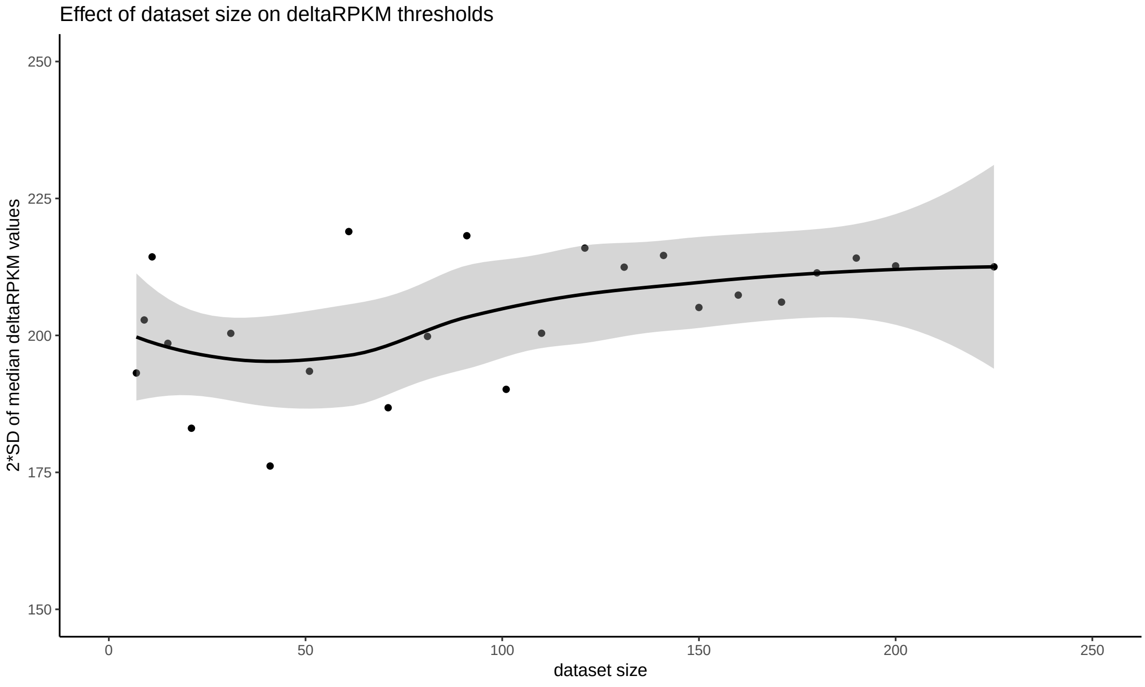

Figure 6: Dataset size effect on the 2*SD(median $\delta RPKM$) thresholding values. The smooth line is built with loess() method; confidence interval in grey.

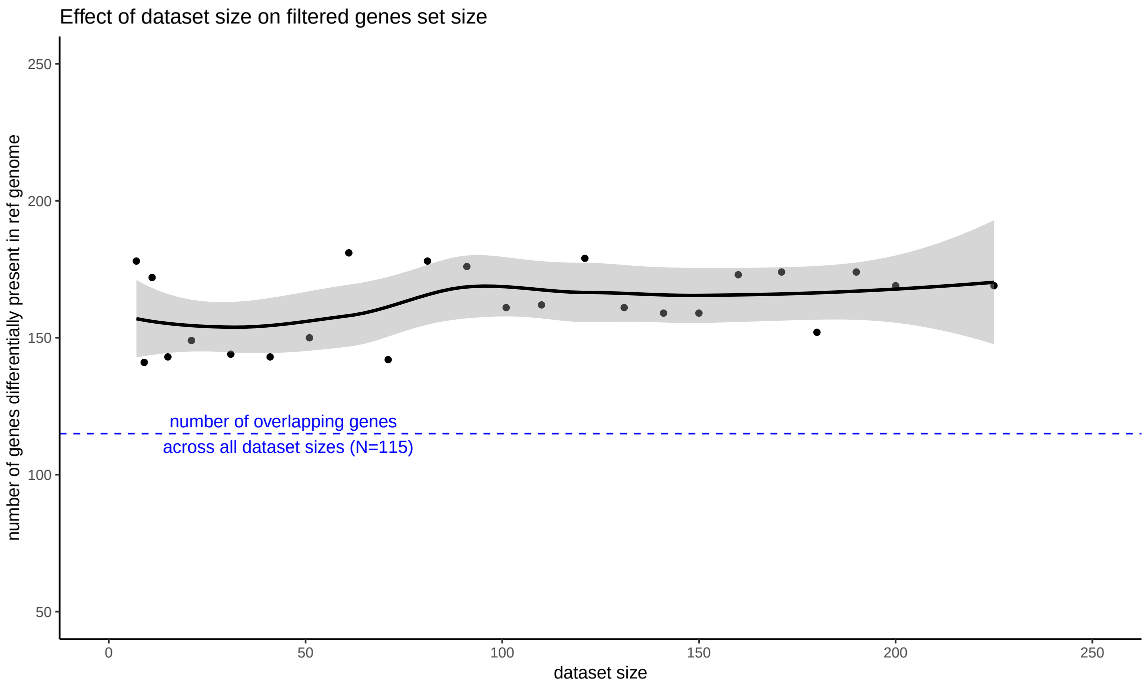

Figure 7: Dataset size effect on genes differentially present in reference genome. The smooth line is built with loess() method; confidence interval in grey.

9.2 Dataset size effect on runtime

The runtime increases linearly with the dataset size (**Figure 8**), but it remains reasonably low.

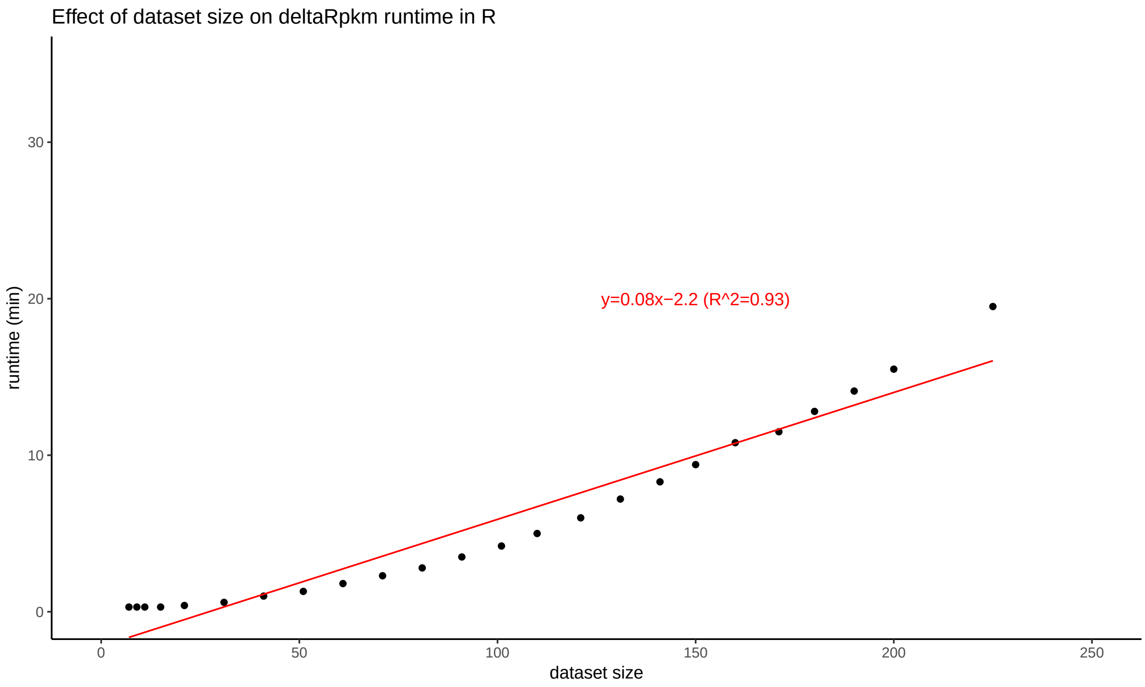

Figure 8: Dataset size and runtime when using deltaRpkm. Including the pipeline steps of RPKM computing, batch effect correction, computing $\delta RPKM$ values, statistics, gene selection and plotting heatmap. Analysing with deltaRpkm a large dataset of N=225 samples takes $<$ **20min** in total in R 3.4.4 (under Ubuntu 14.04.5 LTS).

9.3 Dataset size effect on memory usage

The memory requirement by deltaRpkm analysis grows with the sample size, but in a rather linear way: expect ∼400M every N∼20 samples. So one should be able to run a dataset of up to N∼800 samples on a normal desktop machine with 16G of RAM.

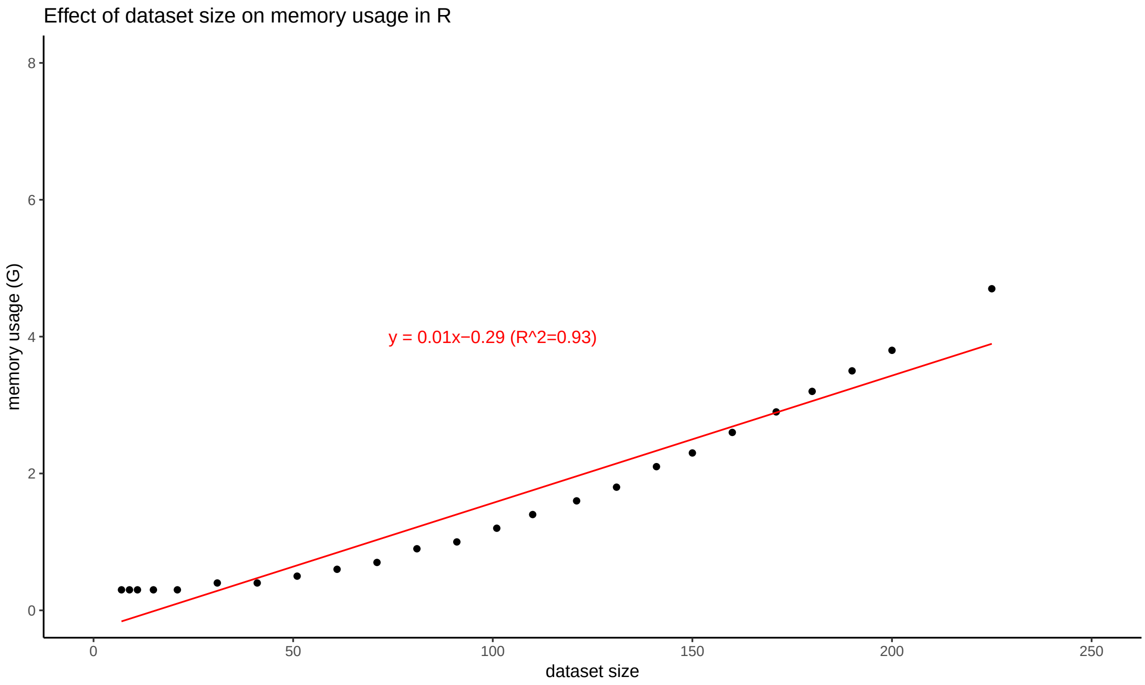

Figure 9: Dataset size and memory usage when using deltaRpkm. The pipeline uses < **4G of memory** for a dataset of N=225 samples, when ran in R 3.4.4 (under Ubuntu 14.04.5 LTS).

9.4 Random datasets

Random datasets confirm the robustness of selected genes with the deltaRpkm method (**Figure 10**). When comparing datasets of different sizes (N=51, N=101, N=225) maintaining the original group classification, most of the genes identified as differentially present in the group 1 are conserved across datasets (**Figure 10(a)**, N=144). While on the other hand, the gene sets derived from random groupings are small and not consistent (**Figure 10(b)**, N=0).

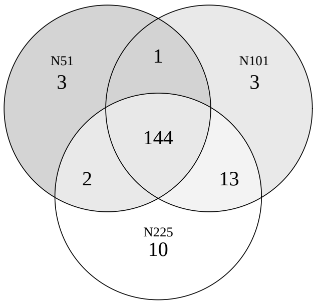

(a) real groups

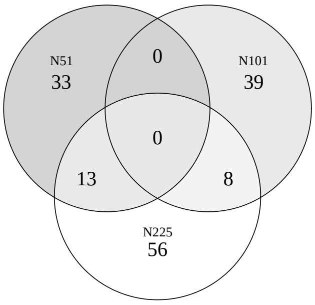

(b) random groups

Figure 10: Real versus random group classification: random group assignment gives non-robust gene sets across different sizes of datasets. deltaRpkm *Listeria monocytogenes* datasets. Batch effect corrected.

10 Binaries and OS platforms

10.1 Ubuntu Trusty Tahr (14.04.5 LTS)

The Linux binary has been built and tested with `R 3.4` under `Ubuntu 14.04 LTS (Trusty Tahr)`:

```
R version 3.4.4 (2018-03-15)
Platform: x86_64-pc-linux-gnu (64-bit)
Running under: Ubuntu 14.04.5 LTS

Matrix products: default
BLAS: /usr/lib/libblas/libblas.so.3.0
LAPACK: /usr/lib/lapack/liblapack.so.3.0

locale:
 [1] LC_CTYPE=en_US.UTF-8       LC_NUMERIC=C               LC_TIME=en_GB.UTF-8        LC_COLLATE=en_US.UTF-8
 [5] LC_MONETARY=en_GB.UTF-8    LC_MESSAGES=en_US.UTF-8    LC_PAPER=en_GB.UTF-8       LC_NAME=C
 [9] LC_ADDRESS=C               LC_TELEPHONE=C             LC_MEASUREMENT=en_GB.UTF-8 LC_IDENTIFICATION=C

attached base packages:
[1] stats     graphics  grDevices utils     datasets  methods   base

other attached packages:
[1] deltaRpkm_0.1.0 ggplot2_3.0.0   bindrcpp_0.2.2  testthat_2.0.0

loaded via a namespace (and not attached):
 [1] colorspace_1.3-2     pryr_0.1.4           colorRamps_2.3       mclust_5.4.1         rprojroot_1.3-2
 [6] XVector_0.16.0       rstudioapi_0.7       roxygen2_6.1.0       bit64_0.9-7          AnnotationDbi_1.38.2
[11] xml2_1.2.0           codetools_0.2-15     splines_3.4.4        annotate_1.54.0      compiler_3.4.4
[16] tictoc_1.0           backports_1.1.2      assertthat_0.2.0     Matrix_1.2-14        lazyeval_0.2.1
[21] limma_3.32.10        cli_1.0.1            tools_3.4.4          gtable_0.2.0         glue_1.3.0
[26] reshape2_1.4.3       dplyr_0.7.6          Rcpp_1.0.0           Biobase_2.36.2       Biostrings_2.44.2
[31] gdata_2.18.0         nlme_3.1-137         stringr_1.4.0        gtools_3.8.1         devtools_1.13.6
[36] XML_3.98-1.16        zlibbioc_1.22.0      scales_1.0.0         parallel_3.4.4       RColorBrewer_1.1-2
[41] memoise_1.1.0        gridExtra_2.3        stringi_1.2.4        RSQLite_2.1.1        genefilter_1.58.1
[46] S4Vectors_0.14.7     desc_1.2.0           caTools_1.17.1.1     BiocGenerics_0.22.1  BiocParallel_1.10.1
[51] rlang_0.3.1          pkgconfig_2.0.2      commonmark_1.6       matrixStats_0.54.0   bitops_1.0-6
[56] lattice_0.20-38      purrr_0.2.5          bindr_0.1.1          labeling_0.3         bit_1.1-14
[61] tidyselect_0.2.4     plyr_1.8.4           magrittr_1.5         R6_2.3.0             IRanges_2.10.5
[66] gplots_3.0.1         DBI_1.0.0            pillar_1.3.0         whisker_0.3-2        withr_2.1.2
[71] mgcv_1.8-27          survival_2.42-6      RCurl_1.95-4.11      ggfortify_0.4.5      tibble_1.4.2
[76] crayon_1.3.4         KernSmooth_2.23-15   grid_3.4.4           sva_3.24.4           data.table_1.11.8
[81] blob_1.1.1           digest_0.6.18        xtable_1.8-3         tidyr_0.8.1          stats4_3.4.4
[86] munsell_0.5.0
```

Figure 11: Session info in R 3.4.4, with RStudio 1.0.143, run under Ubuntu 14.04.5 LTS.

10.2 Ubuntu Bionic Beaver (18.04.2 LTS)

The Linux binary has been built and tested with `R 3.4` under `Ubuntu 18.04.2 LTS (Bionic Beaver)`:

```
R version 3.4.4 (2018-03-15)
Platform: x86_64-pc-linux-gnu (64-bit)
Running under: Ubuntu 18.04.2 LTS

Matrix products: default
BLAS: /usr/lib/x86_64-linux-gnu/blas/libblas.so.3.7.1
LAPACK: /usr/lib/x86_64-linux-gnu/lapack/liblapack.so.3.7.1

locale:
 [1] LC_CTYPE=en_US.UTF-8       LC_NUMERIC=C               LC_TIME=fr_FR.UTF-8
 [4] LC_COLLATE=en_US.UTF-8     LC_MONETARY=fr_FR.UTF-8    LC_MESSAGES=en_US.UTF-8
 [7] LC_PAPER=fr_FR.UTF-8       LC_NAME=C                  LC_ADDRESS=C
[10] LC_TELEPHONE=C             LC_MEASUREMENT=fr_FR.UTF-8 LC_IDENTIFICATION=C

attached base packages:
[1] stats     graphics  grDevices utils     datasets  methods   base

other attached packages:
[1] ggfortify_0.4.5      ggplot2_3.1.0        deltaRpkm_0.1.0      BiocInstaller_1.28.0

loaded via a namespace (and not attached):
 [1] Biobase_2.38.0        pkgload_1.0.2         tidyr_0.8.2           bit64_0.9-7
 [5] splines_3.4.4         gtools_3.8.1          assertthat_0.2.0      stats4_3.4.4
 [9] blob_1.1.1            remotes_2.0.2         sessioninfo_1.1.1     pillar_1.3.1
[13] RSQLite_2.1.1         backports_1.1.3       lattice_0.20-35       glue_1.3.0
[17] limma_3.34.9          digest_0.6.18         RColorBrewer_1.1-2    colorspace_1.4-0
[21] Matrix_1.2-12         plyr_1.8.4            XML_3.98-1.17         pkgconfig_2.0.2
[25] devtools_2.0.1        genefilter_1.60.0     purrr_0.3.0           xtable_1.8-3
[29] scales_1.0.0          gdata_2.18.0          processx_3.2.1        BiocParallel_1.12.0
[33] tibble_2.0.1          annotate_1.56.2       mgcv_1.8-23           IRanges_2.12.0
[37] usethis_1.4.0         withr_2.1.2           BiocGenerics_0.24.0   lazyeval_0.2.1
[41] cli_1.0.1             survival_2.41-3       magrittr_1.5          crayon_1.3.4
[45] memoise_1.1.0         ps_1.3.0              fs_1.2.6              nlme_3.1-131
[49] gplots_3.0.1.1        pkgbuild_1.0.2        tools_3.4.4           prettyunits_1.0.2
[53] matrixStats_0.54.0    stringr_1.4.0         S4Vectors_0.16.0      munsell_0.5.0
[57] AnnotationDbi_1.40.0 callr_3.1.1           compiler_3.4.4        caTools_1.17.1.1
[61] rlang_0.3.1           grid_3.4.4            RCurl_1.95-4.11       rstudioapi_0.9.0
[65] bitops_1.0-6          labeling_0.3          gtable_0.2.0          DBI_1.0.0
[69] reshape2_1.4.3        R6_2.4.0              gridExtra_2.3         dplyr_0.8.0.1
[73] bit_1.1-14            rprojroot_1.3-2       KernSmooth_2.23-15    desc_1.2.0
[77] stringi_1.3.1         parallel_3.4.4        sva_3.26.0            Rcpp_1.0.0
[81] tidyselect_0.2.5
```

Figure 12: Session info in R 3.4.4, with RStudio 1.1.463, run under Ubuntu 18.04.2 LTS.

10.3 MacOS High Sierra (10.13.6)

The MacOS binary has been built and tested with `R 3.4` under `MacOS 10.13.6 (High Sierra)`:

```
R version 3.4.0 (2017-04-21)
Platform: x86_64-apple-darwin15.6.0 (64-bit)
Running under: macOS  10.13.6

Matrix products: default
BLAS: /System/Library/Frameworks/Accelerate.framework/Versions/A/Frameworks/vecLib.framework/Versions/A/libBLAS.dylib
LAPACK: /Library/Frameworks/R.framework/Versions/3.4/Resources/lib/libRlapack.dylib

locale:
[1] en_GB.UTF-8/en_GB.UTF-8/en_GB.UTF-8/C/en_GB.UTF-8/en_GB.UTF-8

attached base packages:
[1] stats     graphics  grDevices utils     datasets  methods   base

other attached packages:
[1] ggfortify_0.4.5 ggplot2_3.1.0   deltaRpkm_0.1.0

loaded via a namespace (and not attached):
 [1] Rcpp_1.0.0          lattice_0.20-38     tidyr_0.8.2         gtools_3.8.1
 [5] assertthat_0.2.0    digest_0.6.18       R6_2.4.0            plyr_1.8.4
 [9] stats4_3.4.0        RSQLite_2.1.1       sva_3.26.0          pillar_1.3.1
[13] gplots_3.0.1.1      rlang_0.3.1         lazyeval_0.2.1      gdata_2.18.0
[17] rstudioapi_0.9.0    annotate_1.56.2     blob_1.1.1          S4Vectors_0.16.0
[21] Matrix_1.2-15       labeling_0.3        splines_3.4.0       BiocParallel_1.12.0
[25] stringr_1.4.0       RCurl_1.95-4.11     bit_1.1-14          munsell_0.5.0
[29] compiler_3.4.0      pkgconfig_2.0.2     BiocGenerics_0.24.0 mgcv_1.8-27
[33] tidyselect_0.2.5    tibble_2.0.1        gridExtra_2.3       IRanges_2.12.0
[37] matrixStats_0.54.0  XML_3.98-1.17       crayon_1.3.4        dplyr_0.8.0.1
[41] withr_2.1.2         bitops_1.0-6        grid_3.4.0          nlme_3.1-137
[45] xtable_1.8-3        gtable_0.2.0        DBI_1.0.0           magrittr_1.5
[49] scales_1.0.0        KernSmooth_2.23-15  stringi_1.3.1       reshape2_1.4.3
[53] genefilter_1.60.0   limma_3.34.9        RColorBrewer_1.1-2  tools_3.4.0
[57] bit64_0.9-7         Biobase_2.38.0      glue_1.3.0          purrr_0.3.0
[61] parallel_3.4.0      survival_2.43-3     AnnotationDbi_1.40.0 colorspace_1.4-0
[65] caTools_1.17.1.1    memoise_1.1.0
```

Figure 13: Session info in R 3.4.0 with RStudio 1.0.143, run under MacOS 10.13.6.

10.4 Windows10

The Windows binary has been built and tested with `R 3.6` under `Windows10`:

```
R version 3.6.1 (2019-07-05)
Platform: x86_64-w64-mingw32/x64 (64-bit)
Running under: Windows >= 8 x64 (build 9200)

Matrix products: default

locale:
[1] LC_COLLATE=French_Switzerland.1252  LC_CTYPE=French_Switzerland.1252
[3] LC_MONETARY=French_Switzerland.1252 LC_NUMERIC=C
[5] LC_TIME=French_Switzerland.1252

attached base packages:
[1] stats     graphics  grDevices utils     datasets  methods   base

other attached packages:
[1] deltaRpkm_0.1.0 testthat_2.2.1  devtools_2.1.0  usethis_1.5.1

loaded via a namespace (and not attached):
 [1] nlme_3.1-140         bitops_1.0-6         matrixStats_0.54.0   fs_1.3.1
 [5] bit64_0.9-7          RColorBrewer_1.1-2   rprojroot_1.3-2      tools_3.6.1
 [9] backports_1.1.4      R6_2.4.0             KernSmooth_2.23-15   DBI_1.0.0
[13] lazyeval_0.2.2       BiocGenerics_0.30.0  mgcv_1.8-28          colorspace_1.4-1
[17] withr_2.1.2          tictoc_1.0           tidyselect_0.2.5     gridExtra_2.3
[21] prettyunits_1.0.2    processx_3.4.1       bit_1.1-14           compiler_3.6.1
[25] cli_1.1.0            Biobase_2.44.0       desc_1.2.0           labeling_0.3
[29] caTools_1.17.1.2     scales_1.0.0         genefilter_1.66.0    callr_3.3.1
[33] stringr_1.4.0        digest_0.6.20        colorRamps_2.3       XVector_0.24.0
[37] pkgconfig_2.0.2      sessioninfo_1.1.1    limma_3.40.6         rlang_0.4.0
[41] rstudioapi_0.10      pryr_0.1.4           RSQLite_2.1.2        mclust_5.4.5
[45] BiocParallel_1.18.1  gtools_3.8.1         dplyr_0.8.3          RCurl_1.95-4.12
[49] magrittr_1.5         Matrix_1.2-17        Rcpp_1.0.2           munsell_0.5.0
[53] S4Vectors_0.22.0     stringi_1.4.3        zlibbioc_1.30.0      pkgbuild_1.0.5
[57] gplots_3.0.1.1       plyr_1.8.4           grid_3.6.1           blob_1.2.0
[61] parallel_3.6.1       gdata_2.18.0         crayon_1.3.4         lattice_0.20-38
[65] Biostrings_2.52.0    splines_3.6.1        annotate_1.62.0      zeallot_0.1.0
[69] ps_1.3.0             pillar_1.4.2         reshape2_1.4.3       codetools_0.2-16
[73] stats4_3.6.1         pkgload_1.0.2        XML_3.98-1.20        glue_1.3.1
[77] data.table_1.12.2    remotes_2.1.0        vctrs_0.2.0          gtable_0.3.0
[81] purrr_0.3.2          tidyr_0.8.3          assertthat_0.2.1     ggplot2_3.2.1
[85] xtable_1.8-4         survival_2.44-1.1    tibble_2.1.3         AnnotationDbi_1.46.1
[89] memoise_1.1.0        IRanges_2.18.2       sva_3.32.1           ggfortify_0.4.7
```

Figure 14: Session info in R 3.6.1, with RStudio 1.1.463, run under Windows10.
